# Supplementary material for: Coordinated maintenance of H3K36/K27 methylation by histone demethylases preserves germ cell identity and immortality
Source: Cell Rep. 2021 Nov 23;37(8):110050. doi: 10.1016/j.celrep.2021.110050 (PMC8640224; doi:10.1016/j.celrep.2021.110050)
Supplement: Document S1. Figures S1–S5 and Table S2 [file mmc1.pdf]

Cell Reports, Volume *37*

Supplemental information

Coordinated maintenance of H3K36/K27 methylation by histone demethylases preserves germ cell identity and immortality

Nico Zaghet, Katrine Madsen, Federico Rossi, Daniel Fernandez Perez, Pier Giorgio Amendola, Samuel Demharter, Ulrich Pfisterer, Konstantin Khodosevich, Diego Pasini, and Anna Elisabetta Salcini

Figure S1

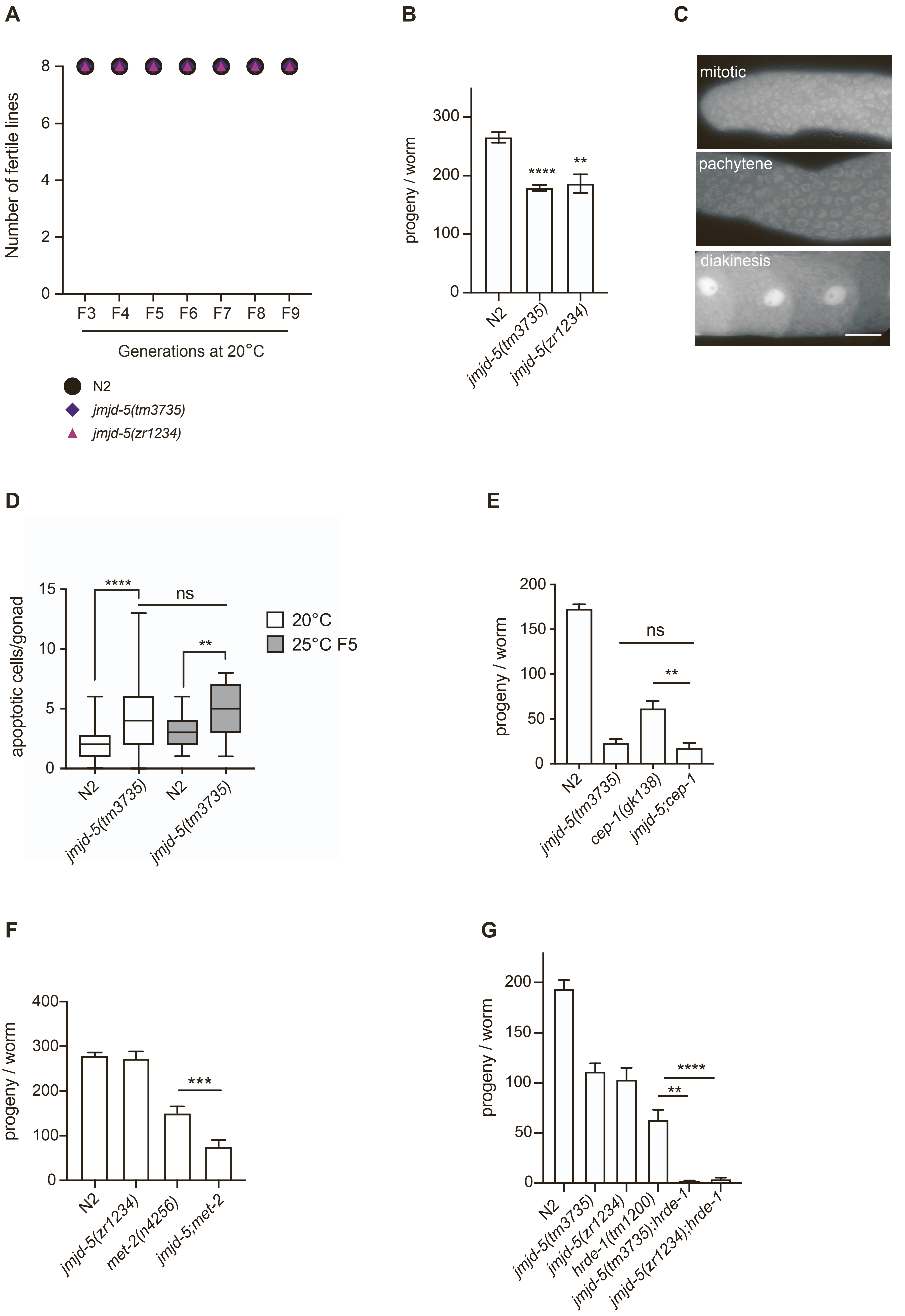
A
B
C
Number of fertile lines
8
6
4
2
0
F3
F4
F5
F6
F7
F8
F9
Generations at 20°C
N2
jmjd-5(tm3735)
jmjd-5(zr1234)
progeny / worm
300
200
100
0
****
**
N2
jmjd-5(tm3735)
jmjd-5(zr1234)
mitotic
pachytene
diakinesis
D
E
apoptotic cells/gonad
15
10
5
0
****
ns
**
20°C
25°C F5
N2
jmjd-5(tm3735)
N2
jmjd-5(tm3735)
progeny / worm
200
150
100
50
0
ns
**
N2
jmjd-5(tm3735)
cep-1(gk138)
jmjd-5;cep-1
F
G
progeny / worm
400
300
200
100
0
***
N2
jmjd-5(zr1234)
met-2(n4256)
jmjd-5;met-2
progeny / worm
200
150
100
50
0
****
**
N2
jmjd-5(tm3735)
jmjd-5(zr1234)
hrde-1(tm1200)
jmjd-5(tm3735);hrde-1
jmjd-5(zr1234);hrde-1

Figure S1. *jmjd-5* preserves the functionality of the germ cells at high temperature, independently of its role in DNA damage response (Related to Fig.1)

(A) Mortal germline assay of *jmjd-5(zr1234)* and *jmjd-5(tm3735)* at 20°C. n=8. (B) Brood size of N2, *jmjd-5(zr1234)* and *jmjd-5(tm3735)* at 15 °C. n>11. (C) Representative image of *jmjd-5::GFP* expression in adult germ cells, detected by direct fluorescence. Bar is 20µm. (D) Boxplot showing the quantification of apoptotic germ cells, using SYTO-12 staining, in N2 and *jmjd-5(tm3735)* grown at 20°C or 25°C for five generations (F5). n>20. (E) Brood size of N2, *jmjd-5(tm3735), cep-1(gk138)* and double mutant grown at 25°C for eight generations (F8). n=20. (F). Brood size of N2, *jmjd-5(zr1234), met-2(n4256)* and double mutants at 20°C. n>10. (G) Brood size of N2, *jmjd-5(zr1234), jmjd-5(tm3735), hrde-1(tm1200)* and double mutants at F1 generation at 25°C. n>10. In B, D-G, bars indicate SEM. ****p<0.0001, ***p<0.001, **p<0.01, ns= not significant, with one-way ANOVA.

Figure S2

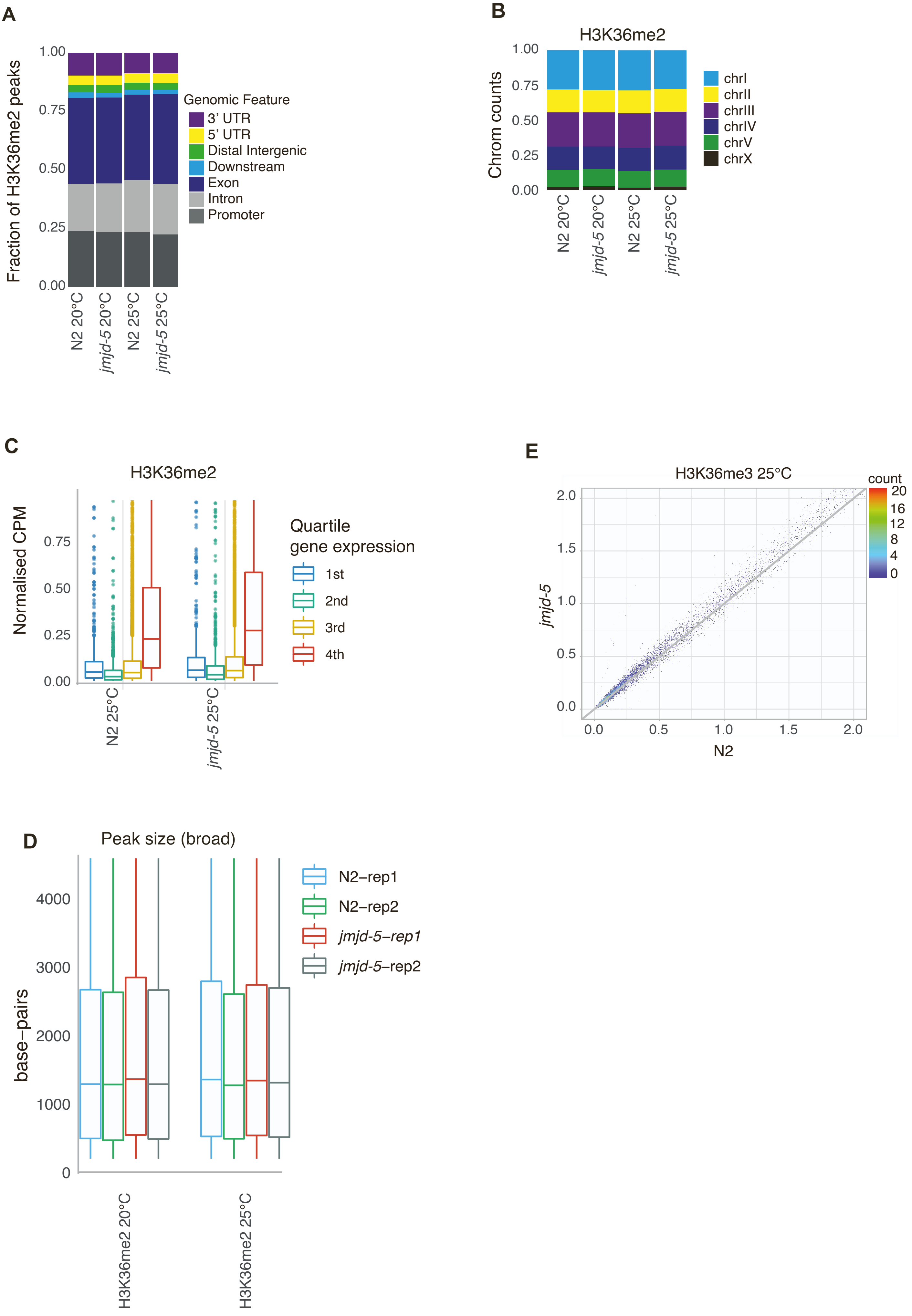
A
Fraction of H3K36me2 peaks
1.00
0.75
0.50
0.25
0.00
N2 20°C
jmjd-5 20°C
N2 25°C
jmjd-5 25°C
Genomic Feature
3' UTR
5' UTR
Distal Intergenic
Downstream
Exon
Intron
Promoter
B
H3K36me2
Chrom counts
chrI
chrII
chrIII
chrIV
chrV
chrX
C
H3K36me2
Normalised CPM
N2 25°C
jmjd-5 25°C
Quartile gene expression
1st
2nd
3rd
4th
E
H3K36me3 25°C
jmjd-5
N2
count
20
16
12
8
4
0
D
Peak size (broad)
base−pairs
4000
3000
2000
1000
0
H3K36me2 20°C
H3K36me2 25°C
N2−rep1
N2−rep2
jmjd-5−rep1
jmjd-5−rep2

Figure S2. H3K36me2/3 ChIP analyses (Related to Fig.4)

(A) Genomic distribution of H3K36me2 peaks in genomic features. Promoters are considered as +/-100bp around TSS, Downstream is considered as > 0 and <1000bp from TES (Transcription End Site) and Distal intergenic +1000bp from TSS (Transcription Start Site). (B) Proportion of H3K36me2 reads aligned to each chromosome in the represented samples. (C) H3K36me2 ChIP-seq signal across all *C. elegans* protein coding genes, divided in 4 quartile groups based on their mRNA expression levels in N2 25°C samples. (D) Peak-size distribution of H3K36me2 ChIP-seq samples. Data for both biological replicates are displayed. (E) Scatterplot of H3K36me3 5kb genome-wide bins signal. N2 vs *jmjd-5(zr1234)* at 25°C is shown.

Figure S3

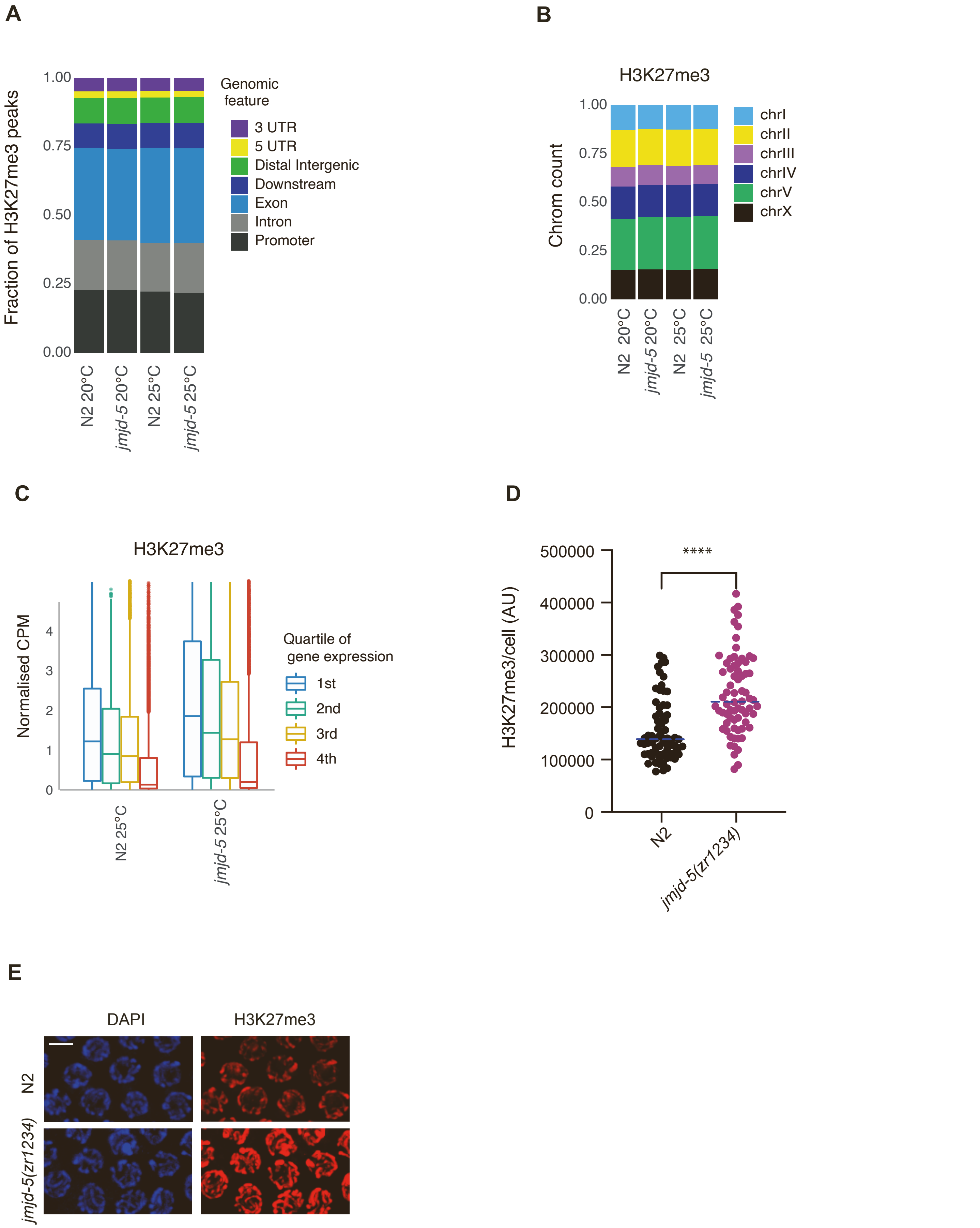
A
Fraction of H3K27me3 peaks
1.00
0.75
0.50
0.25
0.00
N2 20°C
jmjd-5 20°C
N2 25°C
jmjd-5 25°C
Genomic feature
3 UTR
5 UTR
Distal Intergenic
Downstream
Exon
Intron
Promoter
B
H3K27me3
Chrom count
1.00
0.75
0.50
0.25
0.00
N2 20°C
jmjd-5 20°C
N2 25°C
jmjd-5 25°C
chrI
chrII
chrIII
chrIV
chrV
chrX
C
H3K27me3
Normalised CPM
0
1
2
3
4
N2 25°C
jmjd-5 25°C
Quartile of gene expression
1st
2nd
3rd
4th
D
H3K27me3/cell (AU)
500000
400000
300000
200000
100000
0
****
N2
jmjd-5(zr1234)
E
DAPI
H3K27me3
N2
jmjd-5(zr1234)

Figure S3. H3K27me3 ChIP and IF analyses (Related to Fig. 5)

(A) Genomic distribution of H3K27me3 peaks. Promoters are considered as +/-100bp around TSS (Transcription Start Site), Downstream is considered as > 0 and <1000bp from TES (Transcription End Site) and Distal intergenic +1000bp from TSS. (B) Proportion of H3K27me3 reads aligned to each chromosome in the represented samples. (C) H3K27me3 ChIP-seq signal across all *C. elegans* protein coding genes, divided in 4 quartile groups based on their mRNA expression levels in N2 25°C samples. (D) Quantification of the average H3K27me3 intensity (in arbitrary units) per nucleus in pachytene region of N2 and *jmjd-5(zr1234)*. At least 70 cells derived from 7 germlines were quantified for each strain. Lines represent mean. ****$p<0.0001$ with two tailed unpaired t-test. (E) Representative image of H3K27me3 staining in the pachytene region of N2 and *jmjd-5(zr1234)*.

Figure S4

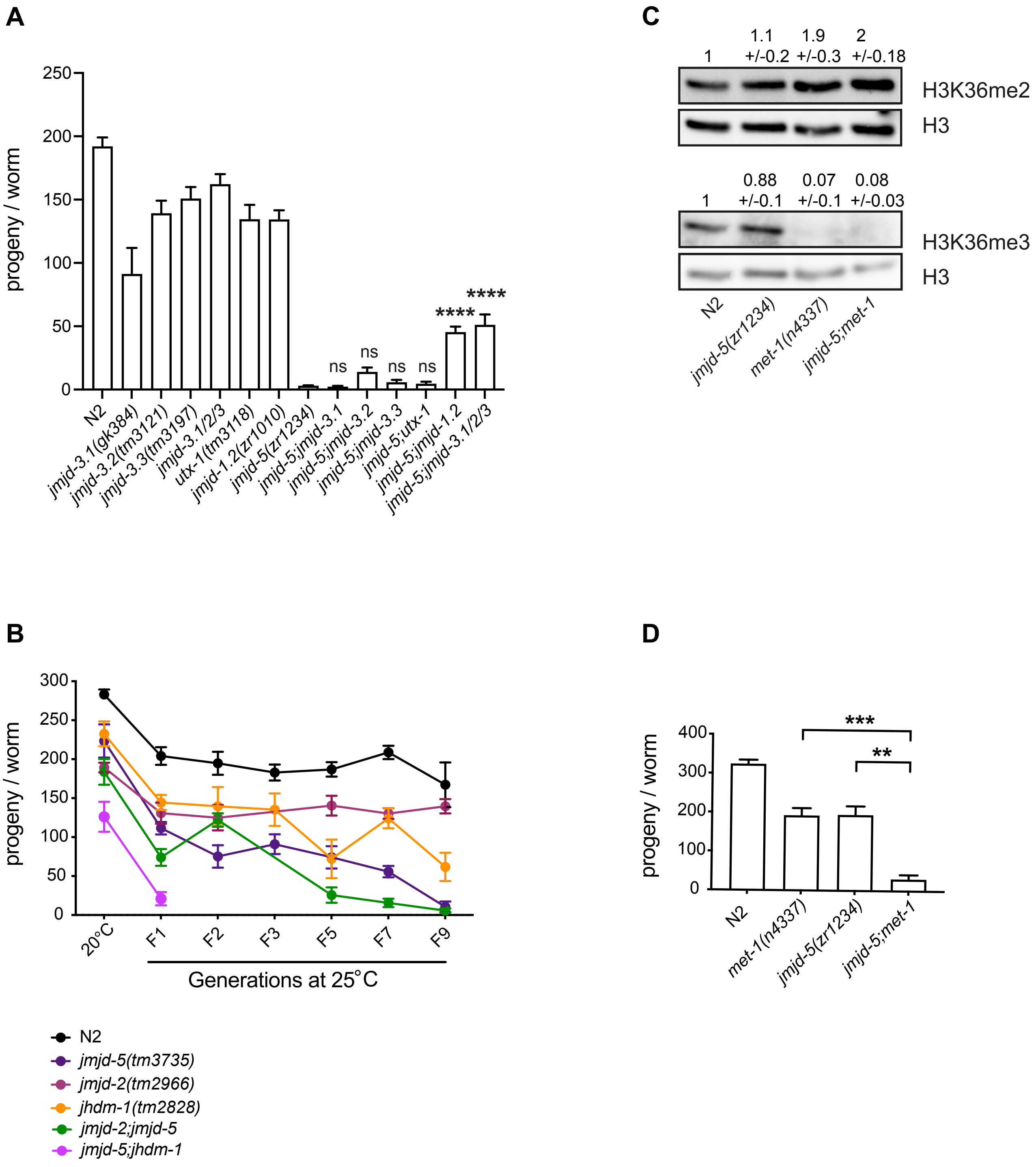

A
progeny / worm
250
200
150
100
50
0
N2
jmjd-3.1(gk384)
jmjd-3.2(tm3121)
jmjd-3.3(tm3197)
jmjd-3.1/2/3
utx-1(tm3118)
jmjd-1.2(zr1010)
jmjd-5(zr1234)
jmjd-5;jmjd-3.1
jmjd-5;jmjd-3.2
jmjd-5;jmjd-3.3
jmjd-5;utx-1
jmjd-5;jmjd-1.2
jmjd-5;jmjd-3.1/2/3
ns
ns
ns
ns
****
****
C
1
1.1 +/-0.2
1.9 +/-0.3
2 +/-0.18
H3K36me2
H3
1
0.88 +/-0.1
0.07 +/-0.1
0.08 +/-0.03
H3K36me3
H3
N2
jmjd-5(zr1234)
met-1(n4337)
jmjd-5;met-1
B
progeny / worm
300
250
200
150
100
50
0
20°C
F1
F2
F3
F5
F7
F9
Generations at 25°C
N2
jmjd-5(tm3735)
jmjd-2(tm2966)
jhdm-1(tm2828)
jmjd-2;jmjd-5
jmjd-5;jhdm-1
D
progeny / worm
400
300
200
100
0
***
**
N2
met-1(n4337)
jmjd-5(zr1234)
jmjd-5;met-1

Figure S4. Genetic interaction of *jmjd-5* with H3K36/K27 methylation regulators (Related to Fig. 7)

(A) Brood size of N2 and indicated mutant strains grown at 25°C for seven generations. Bars represent SEM. n>10. ****p<0.0001, ns, not significative with one-way ANOVA multiple comparison to *jmjd-5(zr1234)*. *utx-1(tm3118)+UTX-1DD::GFP* expresses, under an endogenous promoter, a catalytic-dead version of *utx-1*. (B) Brood size of N2 and indicated mutant strains grown at 20°C and at 25°C, at the indicate generations. (C) Representative western blot and quantified level of H3K36me2/3 (relative to N2), using lysates from synchronized young adult of the indicated strains. H3 is used as loading control. Quantification was performed using two biologically independent replica. (D) Brood size of N2 and indicated mutant strains at 20°C. In A, B and D bars represent SEM. n>15. **** p<0.0001, ***p<0.001, **p<0.01, ns= not significant, with two tailed unpaired t-test.

Figure S5

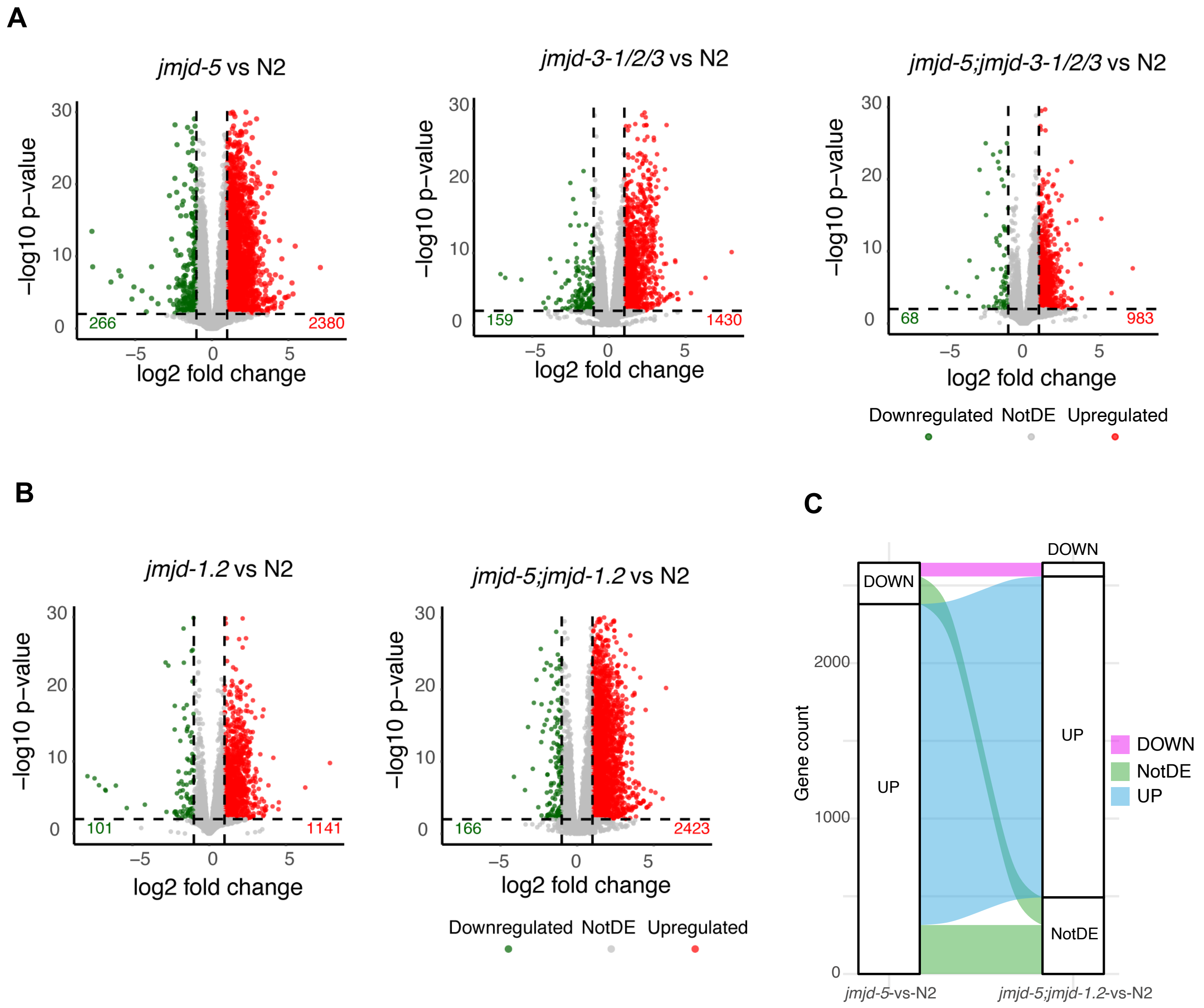
A
jmjd-5 vs N2
jmjd-3-1/2/3 vs N2
jmjd-5;jmjd-3-1/2/3 vs N2
−log10 p−value
log2 fold change
266
2380
159
1430
68
983
Downregulated NotDE Upregulated
B
jmjd-1.2 vs N2
jmjd-5;jmjd-1.2 vs N2
101
1141
166
2423
C
Gene count
DOWN
UP
NotDE
jmjd-5-vs-N2
jmjd-5;jmjd-1.2-vs-N2

Figure S5. Analyses of differentially expressed genes in *jmjd-5(zr1234)*, H3K27 demethylases mutants and compound mutants (Related to Fig. 7).

(A) Volcano plots representing the log2FC and -$\log_{10}$(p-adjusted) of total worm RNA-seq resulting from the following comparisons*: jmjd-5(zr1234)* vs N2 (left*), jmjd-3.1/2/3* vs N2 (center) and *jmjd-5;jmjd-3.1/2/3* vs N2 (right). (B) Volcano plots representing the log2FC and -$\log_{10}$(p-adjusted) of total worm RNA-seq resulting from the following comparisons: *jmjd-1.2* vs N2 (left) and *jmjd-5;jmjd-1.2* vs N2 (right). In A and B, genes with adjusted p-value < 0.01 and log2FC >+/- 1 are considered differentially expressed. (C) Alluvial diagram of differentially expressed genes from *jmjd-5(zr1234)* vs N2 (25°C) and *jmjd-5;jmjd-1.2* vs N2 (25°C).

Table S2. ***jmjd.5*** **prevents germ cells trans-differentiation (Related to Fig. 3)**

Genotype	%P0	%F3	%F4
jmjd-5(zr1234);unc-119::GFP (DP132)	0 (0/64)	18.9 (10/53)	29.4 (15/51)
unc-119::GFP(DP132)	0 (0/30)	0 (0/35)	0 (0/33)
jmjd-5(zr1234);unc-75::GFP(OH443)	0 (0/32)	27.3 (9/33)	35.5 (11/31)
unc-75::GFP(OH443)	0 (0/20)	0 (0/31)	0 (0/30)
jmjd-5(zr1234);lin-17::GFP (KS411)	5.4 (3/56)	33.3 (18/54)	41.9 (14/31)
lin-17::GFP(KS411)	0 (0/31)	0 (0/50)	0 (0/53)

Table S2. Trans-differentiation assay

Extracted germlines of adult animals of the indicated genotypes were analyzed for the presence of GFP fluorescence at the indicated generations at 25°C. n>30
