# Supplementary material for: Coordinated maintenance of H3K36/K27 methylation by histone demethylases preserves germ cell identity and immortality
Source: Cell Rep. 2021 Nov 23;37(8):110050. doi: 10.1016/j.celrep.2021.110050 (PMC8640224; doi:10.1016/j.celrep.2021.110050)
Supplement: Document S2. Article plus supplemental information [file mmc4.pdf]

Cell Reports

Article

Coordinated maintenance of H3K36/K27 methylation by histone demethylases preserves germ cell identity and immortality

Graphical abstract

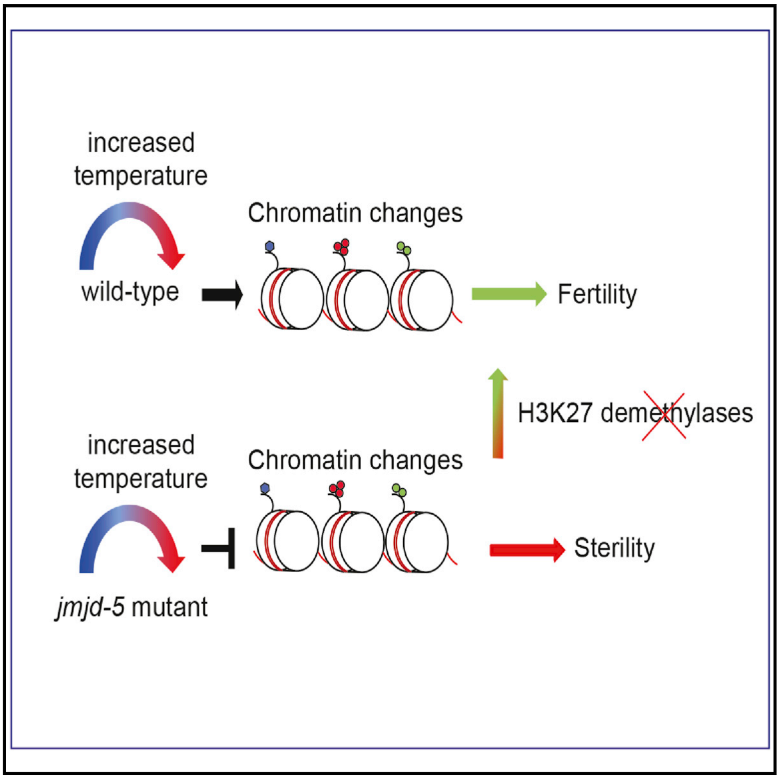

Highlights

- *jmjd-5* is required for germ cell immortality at high temperature
- *jmjd-5* sustains the expression of germline genes and represses somatic fate
- Mutations in *jmjd-5* result in a global increase of H3K36me2 and H3K27me3
- Ablation of H3K27 demethylases counteracts the effects of *jmjd-5* mutations

Authors

Nico Zaghet, Katrine Madsen, Federico Rossi, ..., Konstantin Khodosevich, Diego Pasini, Anna Elisabetta Salcini

Correspondence

lisa.salcini@bric.ku.dk

In brief

Temperature affects reproductive functions in many organisms. Zaghet et al. show that *Caenorhabditis elegans* reacts to temperature rise with chromatin changes, including increase of H3K27 and H3K36 methylation. Mutations in *jmjd-5* prevent these changes and lead to a mortal germline phenotype that is partially rescued by loss of H3K27 demethylases.

Zaghet et al., 2021, Cell Reports *37*, 110050
November 23, 2021 © 2021 The Author(s).
https://doi.org/10.1016/j.celrep.2021.110050

CellPress

Cell Reports

CellPress
OPEN ACCESS

Article

Coordinated maintenance of H3K36/K27 methylation by histone demethylases preserves germ cell identity and immortality

Nico Zaghet,[1] Katrine Madsen,[1] Federico Rossi,[2] Daniel Fernandez Perez,[2] Pier Giorgio Amendola,[1] Samuel Demharter,[1] Ulrich Pfisterer,[1] Konstantin Khodosevich,[1] Diego Pasini,[2,3] and Anna Elisabetta Salcini[1,4,*]
[1]Biotech Research and Innovation Centre (BRIC), University of Copenhagen, Ole Maaløes vej 5, Copenhagen DK-2200, Denmark
[2]Department of Experimental Oncology, IEO European Institute of Oncology IRCCS, Via Adamello 16, 20139 Milan, Italy
[3]Department of Health Sciences, University of Milan, Via A. di Rudini 8, 20142 Milan, Italy
[4]Lead contact
*Correspondence: lisa.salcini@bric.ku.dk
https://doi.org/10.1016/j.celrep.2021.110050

SUMMARY

Germ cells have evolved unique mechanisms to ensure the transmission of genetically and nongenetically encoded information, whose alteration compromises germ cell immortality. Chromatin factors play fundamental roles in these mechanisms. H3K36 and H3K27 methyltransferases shape and propagate a pattern of histone methylation essential for *C. elegans* germ cell maintenance, but the role of respective histone demethylases remains unexplored. Here, we show that *jmjd-5* regulates H3K36me2 and H3K27me3 levels, preserves germline immortality, and protects germ cell identity by controlling gene expression. The transcriptional and biological effects of *jmjd-5* loss can be hindered by the removal of H3K27demethylases, indicating that H3K36/K27 demethylases act in a transcriptional framework and promote the balance between H3K36 and H3K27 methylation required for germ cell immortality. Furthermore, we find that in wild-type, but not in *jmjd-5* mutants, alterations of H3K36 methylation and transcription occur at high temperature, suggesting a role for *jmjd-5* in adaptation to environmental changes.

INTRODUCTION

In multicellular organisms, germ cells carry genetic and epigenetic (not encoded in the DNA) information and represent a continuous cell lineage, connecting all generations. In virtue of this trans-generational link, germ cells are considered as an immortal cell lineage (Furuhashi and Kelly, 2010). Mechanisms preserving germline immortality are vital for the propagation of the species and have been intensively studied in many model organisms, with great contribution from *Caenorhabditis elegans* (*C. elegans*). In *C. elegans*, germ cells originate from two precursor germ cells (PGCs). During larval development, PGCs rapidly proliferate to generate hundreds of germ cells that, if fertilized, will develop to a whole body with different tissues. In addition to self-renewal and totipotency potential, *C. elegans* germ cells are uniquely characterized by the presence of germ granules (P-granules), non-membranous organelles containing proteins and RNAs (Sheth et al., 2010; Updike and Strome, 2010) that protect germ cell identity by preventing aberrant transcription of somatic genes (Knutson et al., 2017; Updike et al., 2014).

Several mechanisms ensure *C. elegans* germ cell immortality, and their inactivation causes a progressive reduction of fertility across generations, which ultimately leads to sterility, a phenotype known as "mortal germline" (Furuhashi and Kelly, 2010; Smelick and Ahmed, 2005). Efficient silencing of repetitive elements and transposons is critical and contributed by small non-coding RNA pathways and heterochromatic factors (Ahringer and Gasser, 2018; Weiser and Kim, 2019). Impaired silencing of repetitive elements leads to genotoxic stress in germline, reduced fertility, and increased apoptosis at high temperature (McMurchy et al., 2017; Zeller et al., 2016). Germ cell immortality is also harmed when cell identity is compromised by unscheduled changes in transcriptional programs, as observed when some chromatin factors are lost. For example, the H3K4 demethylase SPR-5 preserves germline immortality by preventing a progressive accumulation of H3K4me2 in PGCs and aberrant expression of spermatogenic genes (Katz et al., 2009). In contrast, reduced H3K4 methylation by loss of the methyltransferase *set-2* promotes a progressive conversion of the germ cells into somatic-like cells, in concomitance to germline mortality and acquisition of an aberrant epigenetic landscape over generations at high temperature (Robert et al., 2014, 2020). Trans-differentiation of germ cells into more somatic-like fates is also observed when H3K27 methylation is impaired, but with a different modality and timing. Loss of PRC2 complex components, responsible for H3K27 methylation, requires the overexpression of tissue-specific transcription factors to result in an immediate conversion of germ in somatic cells (Patel et al., 2012; Seelk et al., 2016; Tursun et al., 2011). Finally, the analysis of the maternal effect sterility (*mes*)

This is an open access article under the CC BY license (http://creativecommons.org/licenses/by/4.0/).

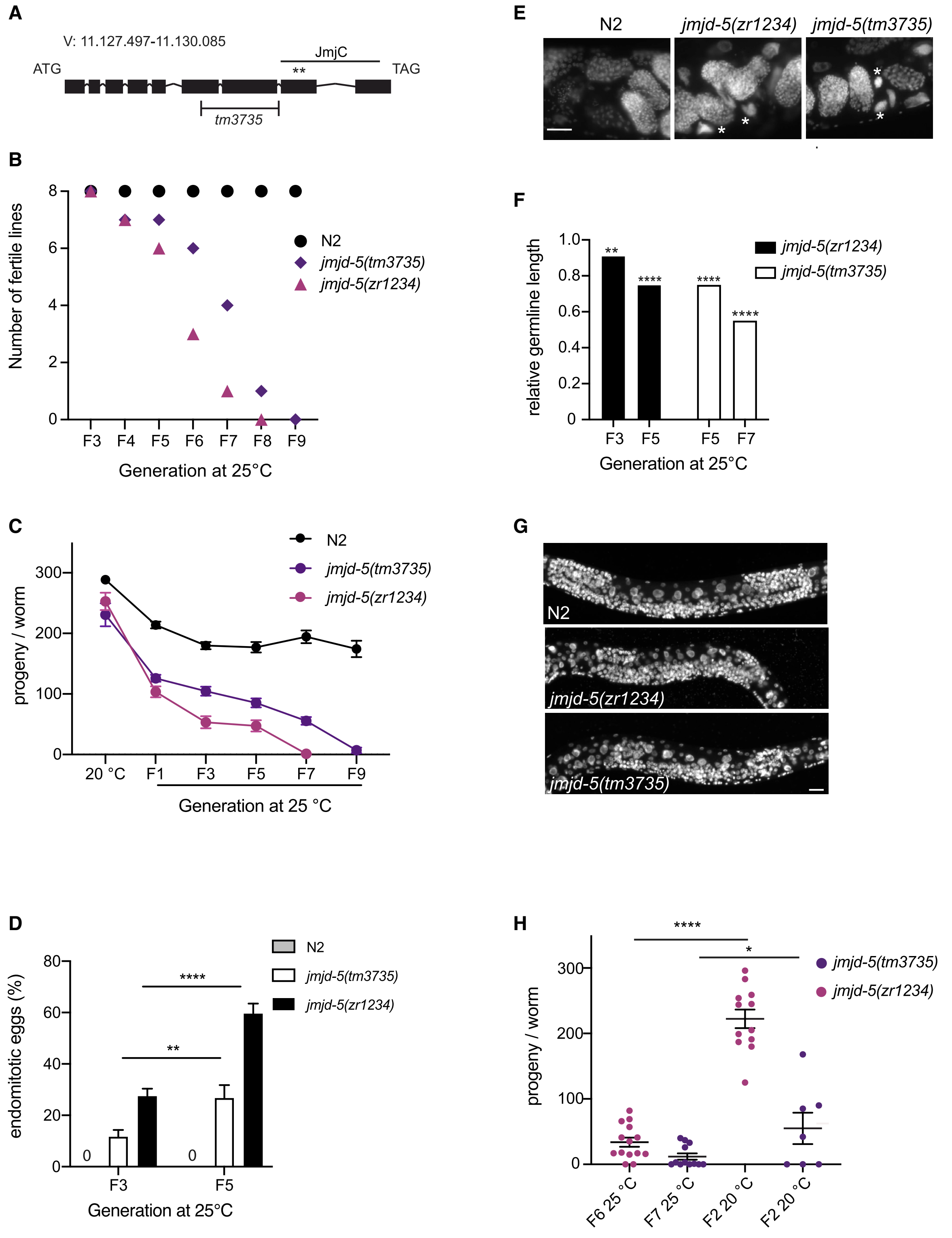

Figure 1. *jmjd-5* mutant germline defects at high temperatures

(A) Genomic structure of *jmjd-5* gene. The H-shaped bar specifies the deletion in *jmjd-5(tm3735)* allele. Asterisks indicate the two amino acids (H484 and D486) mutagenized in *jmjd-5(zr1234)* allele. The position of the JmjC domain is indicated by a black line.

(B) Mortal germline assay at 25°C, using indicated strains. Dots designate the number of fertile lines at the indicated generation. n = 8.

(C) Brood size of indicated strains at 20°C and at indicated generations at 25°C. n > 10.

(legend continued on next page)

phenotype of *mes-4* (a H3K36 methyltransferase) and *mes-2* (a H3K27 methyltransferase) mutants has shown a cross-talk between H3K27 and H3K36 methylation and the relevance of a perfect balance between these marks in promoting immortality and proper transcription in germ cells. *mes-2* and *mes-4* cooperate to support germline gene expression, to repress somatic genes, and to silence X-linked genes (Bender et al., 2004, 2006; Fong et al., 2002; Gaydos et al., 2012; Strome et al., 2014; Tabuchi et al., 2014). Furthermore, studies in embryos showed that *mes-4* is also responsible for the transmission of the memory of germline transcribed genes to the next generation (Furuhashi et al., 2010; Rechtsteiner et al., 2010).

Overall, these findings indicate that chromatin factors impact germ cell immortality, and their role appears particularly relevant, for unknown reasons, when animals are exposed to high temperatures. The involvement of histone demethylases in these processes remains, however, mainly unexplored. In particular, even though severe consequences are observed when H3K36/K27 methylation is reduced in germ cells, the role of corresponding demethylases has not been analyzed.

Here, we report that *jmjd-5* ensures germ cell immortality by protecting germ cell identity at high temperature. Mutations in the JmjC domain of *jmjd-5* result in the increase of H3K36me2 mark and in a concomitant rise of H3K27me3 level. Removal of H3K27 demethylases restores, albeit partially, the fertility of *jmjd-5* mutant animals at high temperature, indicating that a tight modulation of H3K36 and H3K27 methylation mediated by demethylases is required for germline immortality. Finally, we found that in wild-type, but not in *jmjd-5* mutant animals, alterations of H3K36 methylation levels and transcription occur at high temperature, suggesting that *jmjd-5* is required to properly respond to environmental changes.

RESULTS

jmjd-5 is required for sustained fertility at high temperature

While studying the effect of loss of *jmjd-5* in DNA damage response (Amendola et al., 2017), we noticed that *jmjd-5(tm3735)* mutant animals, carrying a large deletion in the *jmjd-5* gene (Figure 1A), showed a temperature-sensitive mortal germline phenotype when cultivated at 25°C (Figure 1B). Analyses of fertility performed on single animals indicated that, when grown at 15°C or 20°C, *jmjd-5(tm3735)* mutant animals show no fertility decline over generations (Figures S1A and S1B). However, when *jmjd-5(tm3735)* mutant animals were grown at 25°C, the number of offspring gradually decreased and, after a few generations, *jmjd-5(tm3735)* mutant animals were sterile (Figure 1C). A similar temperature-sensitive mortal germline phenotype (Figures 1B and 1C) was also observed in *jmjd-5(zr1234)* mutant allele (Amendola et al., 2017), carrying two-point mutations in conserved residues of the JmjC domain (Figure 1A), indicating that the phenotype is neither related to background mutations nor to an intrinsic temperature sensitivity of the mutations and depends on the functionality of the JmjC domain. To further explore the biological consequences of *jmjd-5* loss in germline functions, we analyzed the gonads of mutant animals at different temperatures and generations. When approaching sterility, young adult *jmjd-5* mutant animals showed deteriorated germlines and the presence of endomitotic eggs, whose rate increased with generations at 25°C (Figures 1D and 1E). Furthermore, at early L4 stage, *jmjd-5* gonads were reduced in size, if compared with wild-type, with the germline length progressively reducing across generations (Figures 1F and 1G), suggesting a decreased replicative potential of germ cells during larval development. Consistent with its role in germ cells, JMJD-5 is expressed in the adult gonad (Figure S1C).

We previously showed that *jmjd-5* acts in DNA damage response, and *jmjd-5(tm3735)* animals have an increased level of DNA damage-induced apoptosis, compared with wild-type animals (Amendola et al., 2017) (Figure S1D). We, therefore, wondered whether the phenotype observed at 25°C was due to excessive germ cell apoptosis as a consequence of accumulation of DNA damage over generations. The number of apoptotic corpses was not significantly increased in *jmjd-5(tm3735)* mutants at late generations at 25°C (Figure S1D), and the abrogation of the *cep-1*-induced apoptosis did not rescue the fertility phenotype (Figure S1E), indicating that the mortal germline phenotype is unrelated to checkpoint-dependent apoptosis. This result also suggests that *jmjd-5* is likely not required for repressing repetitive elements, whose aberrant transcription promotes germ cell apoptosis over generations (McMurchy et al., 2017; Zeller et al., 2016). Indeed, when *jmjd-5* was ablated in animals lacking *met-2*, a H3K9 methyltransferase contributing to repetitive element silencing and fertility (Padeken et al., 2021; Zeller et al., 2016), we observed a worsening of the *met-2* fertility phenotype (Figure S1F). Similarly, removal of *jmjd-5* in animals carrying mutations in *hrde-1*, a gene required for retrotransposons silencing and fertility at high temperature (Buckley et al., 2012; Ni et al., 2014), resulted in immediate (F1) sterility at 25°C (Figure S1G). These results suggest that *jmjd-5* is not required for silencing repetitive elements or retrotransposons and acts in parallel to *met-2* and *hrde-1* to protect the animal fertility. In addition, when *jmjd-5* mutant animals grown at 25°C and close to sterility were moved back at lower temperature, they rapidly recovered their fertility (Figure 1H), indicating that this phenotype is reversible and therefore unrelated to an accumulation of gene mutations over

(D) Percentage of germlines with endomitotic eggs. Data are from three biological replicas. n > 95.

(E) Representative images of DAPI-stained endomitotic eggs, marked by asterisks, in animals of the indicated genotypes grown at 25°C for five generations. Scale bar: 20 μm.

(F) Length of germlines (normalized to N2) at early L4 stage, measured in *jmjd-5* mutants grown at 25°C at the indicated generations. n > 17.

(G) Representative images of DAPI-stained germlines of N2 and *jmjd-5* mutants grown at 25°C for five generations. Scale bar: 10 μm.

(H) Reversibility of the mortal germline phenotype. *jmjd-5(zr1234)* 25°C: n = 14; *jmjd-5(tm3735)* 25°C: n = 12; *jmjd-5(zr1234)* 20°C: n = 12; *jmjd-5(tm3735)* 20°C: n = 7. Data are obtained from two biological replica.

In (C), (D) and (H), bars indicate SEM. ****p < 0.0001, **p < 0.01, *p < 0.05, with two-tailed, unpaired t test. See also Figure S1.

generations. Notably, mortal germline reversibility and no increased DNA damage are features observed also in small RNA pathways mutants (Reed et al., 2020; Rogers and Phillips, 2020; Spichal et al., 2021), suggesting that these aspects could be a hallmark of the temperature-sensitive mortal germline phenotype controlled by different pathways.

Overall, these results point to a role of *jmjd-5* in preserving the functionality of the germ cells at high temperature, independently of its role in DNA damage repair.

JMJD-5 regulates transcription in germ cells

To test the transcriptional effect of *jmjd-5* mutations specifically in germ cells, we performed RNA sequencing of isolated germlines. Dissected germlines of wild-type and *jmjd-5(zr1234)* animals grown at 15°C and after three generations at 25°C (F3-25°C), when the gonads have only a minor reduction in size (Figure 1F) and can be easily extracted, were used. We identified 10,561 genes expressed in wild-type gonads at 15°C (cut-off of $\geq$2 reads/gene in average; Table S1). This list of genes largely overlaps (81.2%, 8,727/10,754) with a published dataset of germline transcripts identified by a similar technical approach using multiple gonads (Ortiz et al., 2014). Comparison of our dataset with the list of mRNAs detected by a SAGE approach for genes expressed in the germline (Wang et al., 2009) found a strong match (92.9%, 4,636/4,699), which was further increased (96.4%, 1,026/1,063) when genes enriched in the germline were considered. This evidence indicates that the RNA sequencing data obtained using single extracted germlines are representative of the germline transcriptome.

The comparison of the transcriptome of germline extracted from wild-type and *jmjd-5* mutant animals grown at 15°C identified only 383 differentially expressed (DE) genes ($p \leq 0.01$) (Table S1), of which 94 upregulated and 289 downregulated in *jmjd-5* mutant animals. When the transcriptome of *jmjd-5(zr1234)* mutant animals grown for three generations at 25°C was compared with the one of wild-type animals raised in similar conditions, we found 736 DE genes ($p \leq 0.01$), of which 336 downregulated and 400 upregulated in *jmjd-5* mutant animals (Table S1). Of note, the overlap between *jmjd-5* DE genes at 15°C and 25°C was minimal, with only 33 genes DE in common, suggesting that *jmjd-5* affects, directly or indirectly, the transcription of specific targets in a temperature-sensitive manner. Gene Ontology (GO) analyses of the 736 DE genes highlighted categories related to reproduction, germ cell development, cell division, and, surprisingly, nervous system development (Figure 2A). We further analyzed the *jmjd-5* DE genes at 25°C by categorizing them into germline expressed and germline enriched, based on published datasets (Wang et al., 2009). Downregulated genes were over-represented, while upregulated genes were under-represented, in the germline-expressed and germline-enriched groups (Figure 2B). Of note, more than 10% (45/400) of the upregulated genes in *jmjd-5* mutant animals at 25°C were exclusively expressed in somatic tissues in wild-type animals (Table S1). The visualization of the chromosomal distribution of *jmjd-5(zr1234)* DE genes, at 15°C and 25°C, revealed that the increase of temperature affects mainly the X chromosome expression (Figure 2C), with a 4-fold increase of X-linked genes downregulated at 25°C compared with 15°C. Interestingly, more than half (34/62) of the X-linked downregulated genes are described as oogenic.

Overall, these results suggest that *jmjd-5* controls germ cell transcription by sustaining the expression of germline genes and by repressing the expression of somatic genes. Furthermore, *jmjd-5* has a role on X chromosome activation specifically at high temperature and contributes to the expression of the few germline genes located in the X chromosome.

Loss of *jmjd-5* leads to a progressive loss of germ cell identity at high temperature

To validate the RNA sequencing results obtained from isolated germlines, we selected *unc-75* and *lin-17* among the list of somatic genes (Table S1) found aberrantly expressed in the germline of *jmjd-5* animals grown at 25°C, and we tested their expression using GFP-transgenic lines. We did not detect GFP expression in wild-type germlines at any temperature tested or in *jmjd-5* at 15°C (Table S2; Figure 3A). However, when *jmjd-5(zr1234)* animals were cultivated at 25°C, GFP expression become evident in the germ cells with a rate increasing with the number of generations (Table S2; Figure 3A). Similar results were obtained with a *jmjd-5(zr1234)* line carrying the pan-neuronal marker *unc-119::GFP* (Table S2). The disordered anatomical structure of the mutant gonads at late generations prevented us from clearly identifying whether the GFP expression started in mitotic cells or occurred in the meiotic compartment; nevertheless, this result strongly suggests that germ cells of mutant animals grown for some generations at high temperature assume somatic-like fates.

In several organisms, one of the hallmarks of germ cells is the presence of germ granules (P-granules in *C. elegans*). In *C. elegans* embryo, P-granules are exclusively present in precursor germ cells (PGCs), with a reproducible pattern of localization surrounding the nucleus (Updike and Strome, 2010). To test whether *jmjd-5* PGCs have normal P-granules structures, wild-type and *jmjd-5(zr1234)* mutant animals were grown at 15°C and 25°C for two generations, and F3 embryos were stained using a specific antibody recognizing PGL-1, a core component of P-granules (Updike and Strome, 2010). We observed a reduction of PGL-1 in PGCs in *jmjd-5(zr1234)* at 15°C, compared with N2, that is exacerbated after three generations at 25°C (Figures 3B and 3C). Our experimental setting revealed also a reduction of PGL-1 signal in wild-type animals at 25°C, compared with 15°C, as previously observed and related to the liquid-like properties of P-granules (Brangwynne et al., 2009; Seydoux, 2018). Despite a considerable reduction of these cellular structures, P-granules components are not on the list of downregulated genes in *jmjd-5* mutant animals grown at high temperature, suggesting that post-transcriptional mechanisms (Marnik et al., 2019) might contribute to the reduction of P-granules in *jmjd-5* animals. Intriguingly, similar decrease of P-granules is observed in small RNA pathway mutant exhibiting a temperature-dependent mortal germline (Rogers and Phillips, 2020). However, our genetic data (Figure S1G) indicate that *jmjd-5* acts in parallel of *hrde-1*, a main factor in the nuclear small RNA machinery, suggesting that multiple parallel pathways are likely contributing to P-granules content. Several reports indicate that loss of P-granules can lead to trans-differentiation of germ cells and to

CellPress
OPEN ACCESS

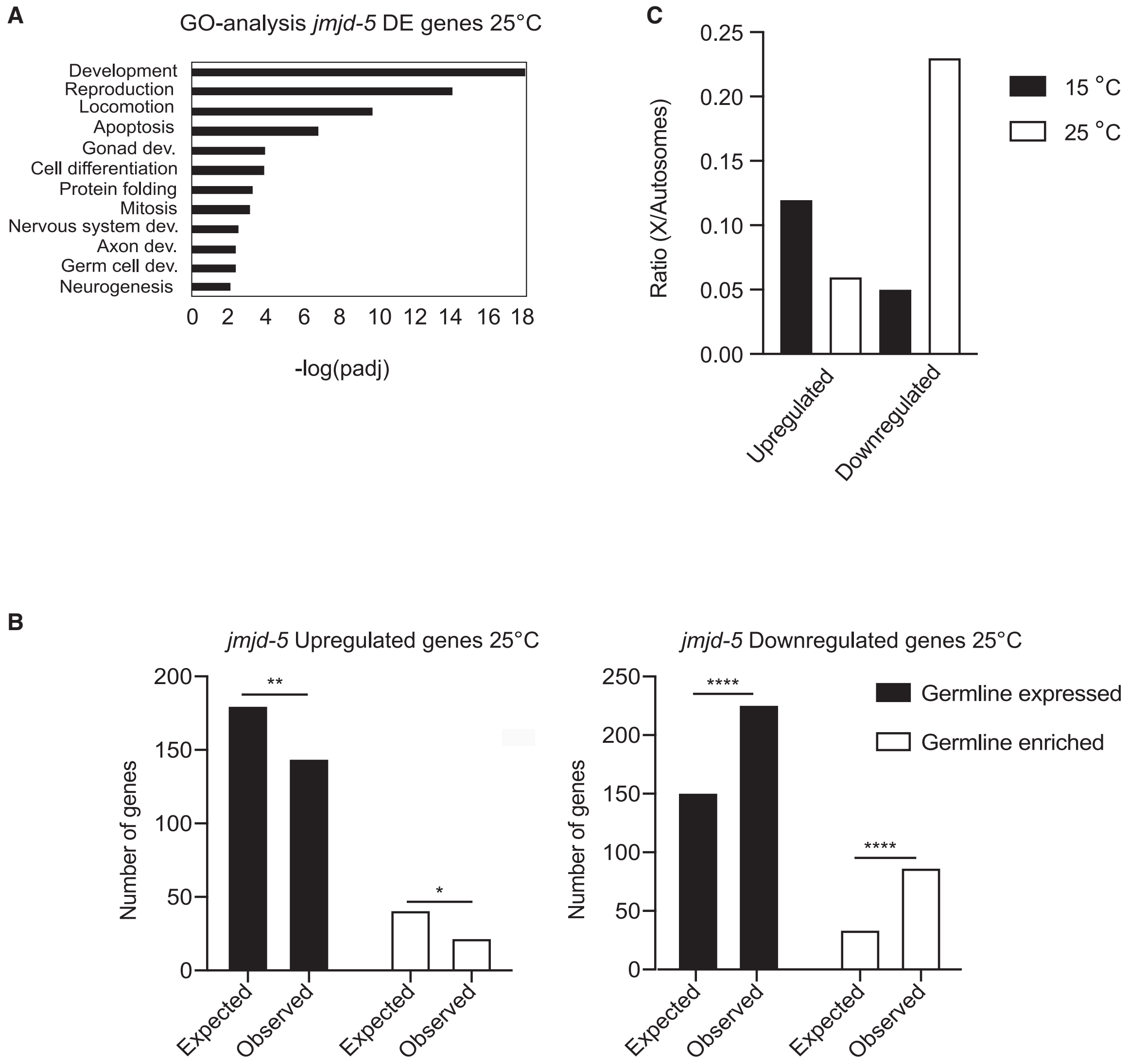

Figure 2. *jmjd-5* controls transcription in germ cells

(A) Gene Ontology (GO) analysis of differentially expressed (DE) genes in *jmjd-5(zr1234)* mutant animals identified by comparing the germline transcriptomes of N2 and *jmjd-5(zr1234)* animals grown for three generations at 25°C ($p < 0.01$). Selected GO terms are presented as $-\log_{10}$(p-adjusted).

(B) Expected and observed number of germline-expressed and germline-enriched genes in upregulated (left) and downregulated (right) genes identified as in (A). ****$p < 0.0001$, **$p < 0.01$, *$p < 0.05$ with Fisher's exact test.

(C) Chromosomal distribution of *jmjd-5(zr1234)* DE genes identified at 15°C and 25°C, compared with N2 animals raised in similar conditions ($p < 0.01$). The graphic reports the ratio between *jmjd-5(zr1234)* DE genes at 15°C and 25°C, located on the X chromosome and autosomes.

transcriptome changes (Knutson et al., 2017; Updike et al., 2014). Still, we did not find a significant overlap ($p = 0.09$) among the lists of genes deregulated in *jmjd-5* and *pgl-1* mutants, suggesting that the P-granules defects observed in *jmjd-5(zr1234)* are a consequence rather than the cause of the *jmjd-5* mortal germline. All together, these results suggest that *jmjd-5* germ cells have a progressive loss of germ cell identity, which is likely accountable for the defective fertility observed at high temperature.

JMJD-5 regulates H3K36me2 level

Previous analyses (Amendola et al., 2017) indicated that JMJD-5 regulates the level of H3K36me2. To further investigate the contribution of *jmjd-5* to H3K36me2 distribution and level, we performed H3K36me2 chromatin immunoprecipitation (ChIP), probing chromatin extracted from whole young adult wild-type and *jmjd-5(zr1234)* animals. Consistent with previous analyses (Evans et al., 2016; Rechtsteiner et al., 2010), we found that H3K36me2, in wild-type animals grown at 20°C, is particularly enriched in genic regions (Figure S2A), widely distributed on autosomes, almost depleted from the X chromosome (Figure S2B), and mainly present in actively transcribed genes (Figure S2C). A similar pattern of H3K36me2 was observed in *jmjd-5(zr1234)* mutants, indicating that mutations in *jmjd-5* do not impact the overall distribution and localization of H3K36me2 (Figures S2A–S2C). The size of

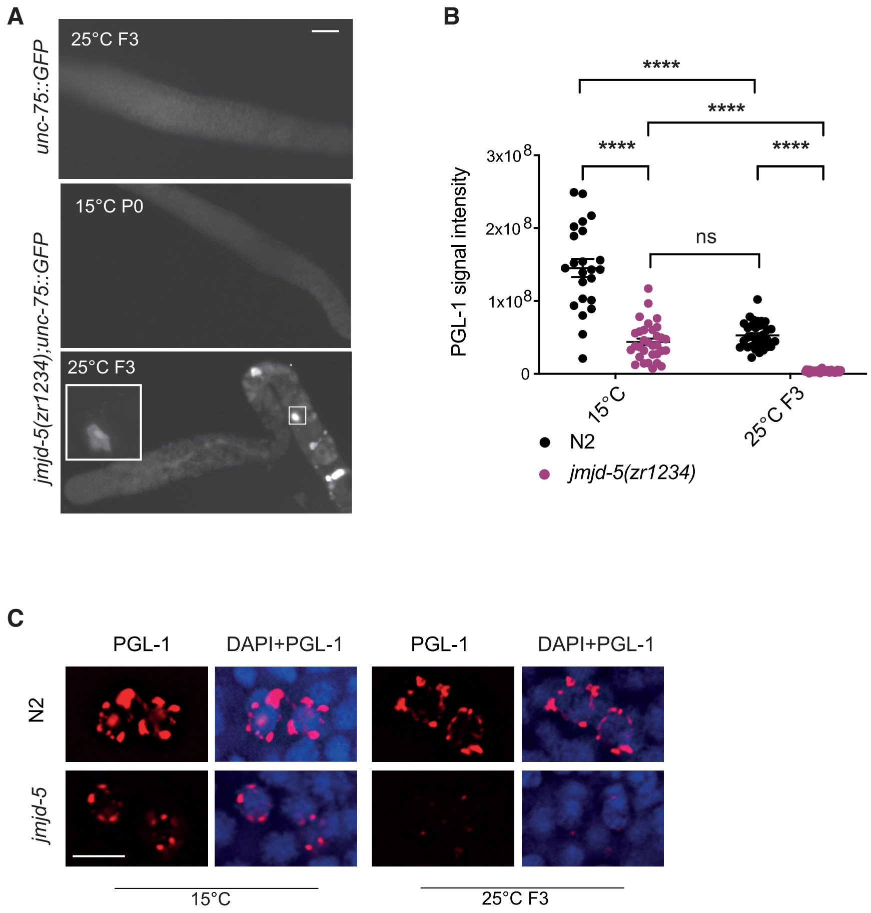

Figure 3. *jmjd-5* protects germ cells from trans-differentiation at high temperature

(A) Representative images of GFP expression in extracted germlines of *unc-75::GFP* and *jmjd-5(zr1234);unc-75::GFP* grown at 15°C or at 25°C at indicated generations. A magnification of a GFP-positive cell is shown in the square. Scale bar: 50 μm. Genotypes and numbers of germlines analyzed are presented in Table S2.

(B) Quantification of PGL-1 signal in PGCs of N2 and *jmjd-5(zr1234)* at the indicated temperature and generations. Each dot represents an embryo ($n > 23$). ****$p < 0.0001$, with one-way ANOVA.

(C) Representative images N2 and *jmjd-5(zr1234)* PGCs, from animals grown at the indicated temperatures and generations, stained with PGL-1 and DAPI. Scale bar: 5 μm.

ns, not significant.

the H3K36me2 peaks was also unchanged in *jmjd-5(zr1234)*, excluding a role of *jmjd-5* in constraining H3K36me2-positive regions (Figure S2D). Nonetheless, the level of H3K36me2 increased throughout the genome in *jmjd-5(zr1234)* animals, compared with wild-type animals. We measured H3K36me2 at enriched sites (peaks) in wild-type and *jmjd-5(zr1234)* mutant and found a large overlap among them (Figure 4A) and a H3K36me2 increased level in *jmjd-5(zr1234)* mutant, both at 20°C and 25°C (Figures 4B and 4C). To extend this observation, we further divided the entire genome in 5-kb windows and demonstrated that H3K36me2 levels increased at all genomic sites, including at regions with low basal levels of H3K36me2 (Figure 4D). Together, these data indicate that *jmjd-5* is devoted to regulating H3K36me2 accumulation in regions that are normally marked by this modification but is not involved in determining its specific genomic localization.

When H3K36me2 ChIP sequencing (ChIP-seq) analyses were performed using material obtained from whole animals grown at 25°C for three generations, we again observed an overall increase of H3K36me2 levels in *jmjd-5* animals compared with wild-type at the same temperature (Figures 4A–4D; Figures S2A–S2D). However, we did not observe a further increase of H3K36me2 when we compared the level at the two temperatures in *jmjd-5* mutant animals (Figure 4B). This lack of increase of H3K36me2 at high temperature is apparently not due to a rapid conversion of H3K36me2 to H3K36me3, because ChIP for H3K36me3 in wild-type and *jmjd-5* mutant animals grown at 25°C for three generations showed no changes in H3K36me3 level (Figure S2E).

These results confirm the role of *jmjd-5* in maintaining proper H3K36me2 global level and are consistent with a putative H3K36me2 demethylase activity of JMJD-5.

jmjd-5 loss correlates with increased H3K27me3 level

Multiple studies have illustrated the cross-talk between H3K27 and H3K36 methylation in many organisms, including *C. elegans* (Finogenova et al., 2020; Gaydos et al., 2012; Streubel et al., 2018), and several observations suggest that *jmjd-5* might contribute to it. First, the impact of *jmjd-5* loss at 25°C in germline transcription (downregulation of germline genes and increased expression of somatic genes) is reminiscent of the effect of loss of *mes-2*/*mes-4* (Gaydos et al., 2012; Patel et al., 2012). Second, similar to *mes* genes, in germ cells, *jmjd-5* regulates the expression pattern of the X chromosome, mainly by promoting gene expression (62 X-linked genes are downregulated and 23 are upregulated at 25°C in *jmjd-5* mutants), with a transcriptional effect that is the opposite of the one observed in the *mes* mutants. Finally, *jmjd-5* regulates the expression of some *mes* targets (Seelk et al., 2016; Tabuchi et al., 2014) (Table S1).

To verify whether *jmjd-5* contributes to the H3K36/K27 methylation cross-talk, we analyzed the genome-wide localization for H3K27me3 by ChIP in *jmjd-5(zr1234)* mutant animals grown at 20°C and 25°C for three generations. In wild-type animals, H3K27me3 was widely distributed on genomic features, including intergenic regions, and was abundant on the X chromosome (Figures S3A and S3B), with preferential accumulation at repressed genes (Figure S3C) at both temperatures. A similar distribution pattern was observed in *jmjd-5(zr1234)* mutant animals, indicating that mutations in *jmjd-5* do not impact H3K27me3 localization. Nevertheless, *jmjd-5(zr1234)* mutant

CellPress
OPEN ACCESS

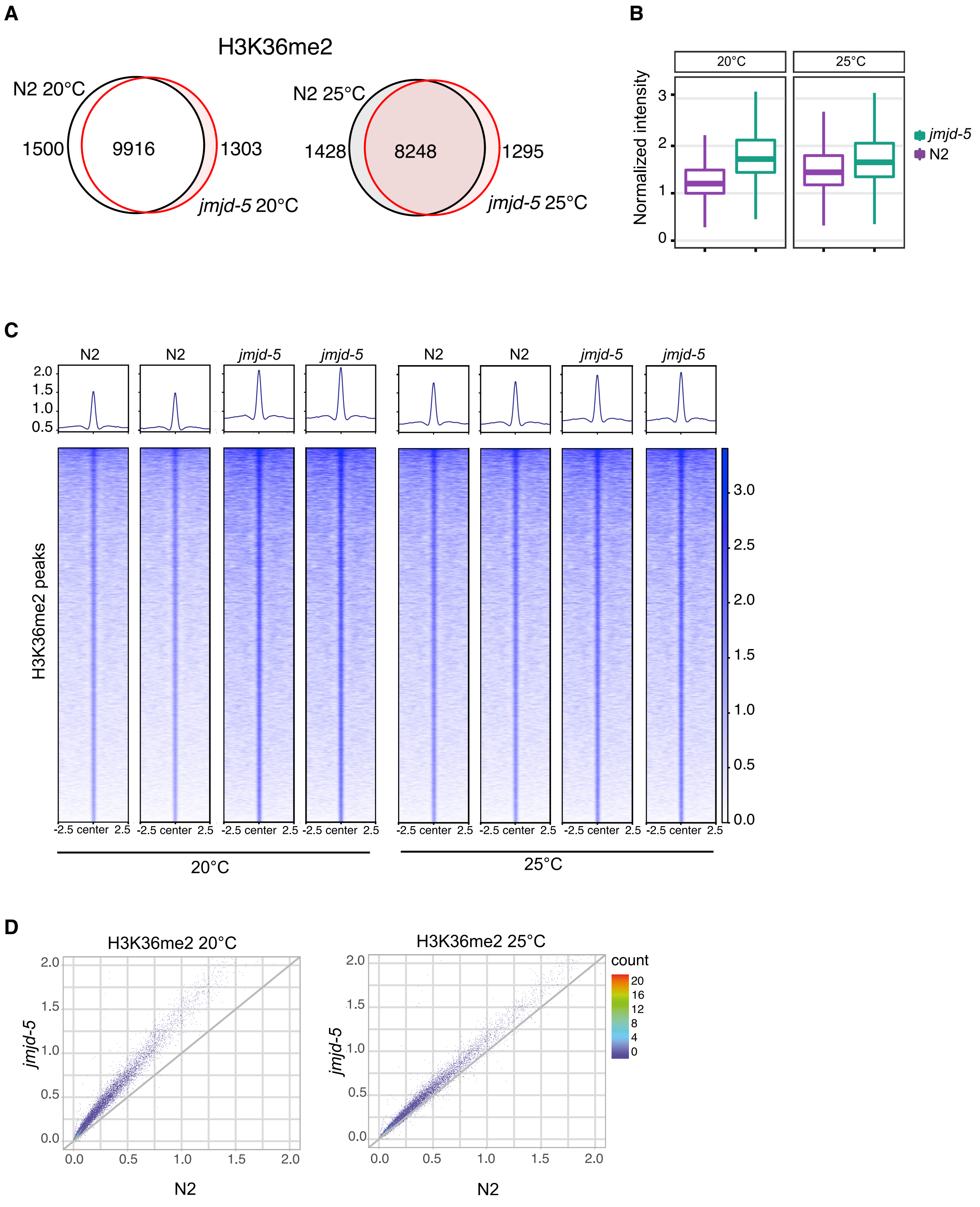

Figure 4. ***jmjd-5*** **controls H3K36me2 levels across the entire genome**

(A) Venn diagram of H3K36me2 peaks. Left: 20°C (N2 versus *jmjd-5*(*zr1234*)); right: 25°C (N2 versus *jmjd-5(zr1234)*).

(B) Boxplots of normalized H3K36me2 ChIP-seq signal across H3K36me2 consensus peaks of N2 samples and *jmjd-5* mutants at 20°C (left) and 25°C (right).

(C) Heatmaps of H3K36me2 ChIP-seq signal across H3K36me2 consensus peaks in N2 and *jmjd-5(zr1234)* at 20°C and 25°C. Two replica are shown.

(D) Scatterplots of H3K36me2 5-kb genome-wide bins signal. N2 versus *jmjd-5(zr1234)* at 20°C (left) and N2 versus *jmjd-5(zr1234)* at 25°C (right).

See also Figure S2.

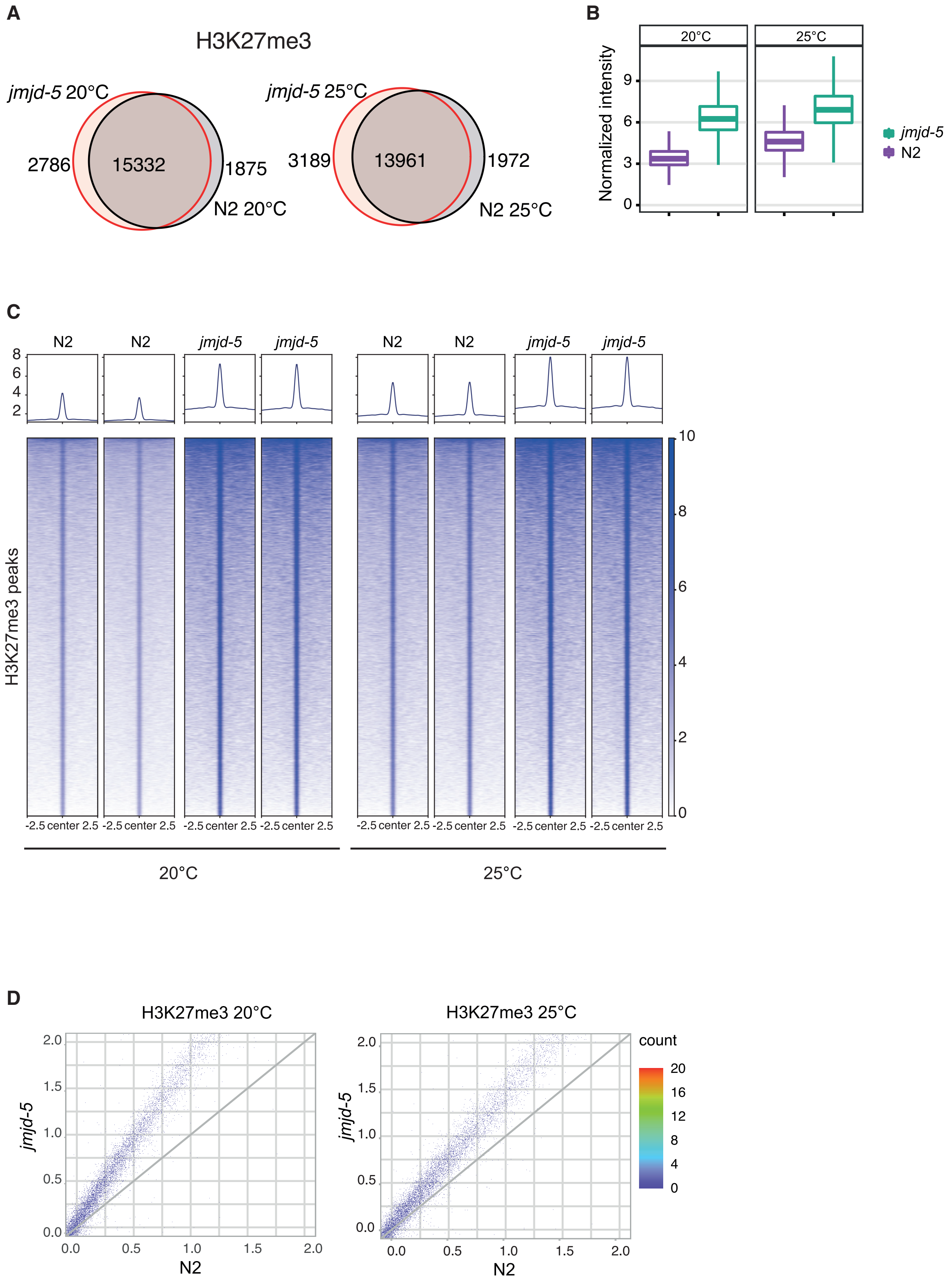

Figure 5. *jmjd-5* regulates the cross-talk with H3K27me3

(A) Venn diagram of H3K27me3 peaks. Left: 20°C (N2 versus *jmjd-5(zr1234)*); right: 25°C (N2 versus *jmjd-5(zr1234)*).

(B) Boxplots of normalized H3K27me3 ChIP-seq signal across H3K27me3 consensus peaks of N2 samples and *jmjd-5* mutants at 20°C (left) and 25°C (right).

(legend continued on next page)

animals showed a substantial increase in global H3K27me3 levels with respect to wild-type. Similar to H3K36me2-enriched regions, this mainly occurred at loci that are H3K27me3 enriched in wild-type animals (Figure 5A). All the enriched loci accumulated H3K27me3 levels in the absence of *jmjd-5* mutant, both at 20°C and 25°C (Figures 5B and 5C). This was further confirmed by dividing the genome in 5-kb regions (Figure 5D). This analysis showed H3K27me3 levels increased globally across the entire chromatin, regardless of its basal enrichments. Interestingly, the enhancement of H3K27me3 level observed at 20°C was slightly amplified at 25°C (Figure 5B). Immunofluorescence approaches confirmed that H3K27me3 level is increased in *jmjd-5* germ cells (Figures S3D and S3E).

Thus, the modulation of H3K36me2 in *jmjd-5* mutant animals is accompanied by a genome-wide increase of H3K27me3, indicating that *jmjd-5* contributes to the cross-talk between H3K36 and H3K27 methylation.

Temperature effects on H3K36/27 methylation and transcription

Both H3K27me3 and H3K36me2 are preferentially deposited within gene bodies (Figures S2A and S3A). To determine whether positive and negative correlations exist, we used the ChIP-seq signals from gene bodies for both H3K27me3 and H3K36me2 in wild-type animals to perform unsupervised K-mean clustering (Figure 6A). This allowed to identify five distinct gene clusters with specific H3K36me2 and H3K27me3 patterns. The analysis showed that the distribution of the marks along the gene bodies is unchanged in *jmjd-5* mutant animals, compared with wild-type, at both temperatures (Figure 6B) and confirmed that H3K36me2 and H3K27me3 are mutually exclusive, and no cluster with high levels for both modifications was identified (Figures 6A and 6B). Instead, we found two gene clusters with high (C5) and moderate (C3) H3K36me2 levels, respectively, both presenting low levels of H3K27me3. An additional two clusters showed an opposite pattern, with high (C4) or moderate (C2) H3K27me3 levels, respectively, and low H3K36me2 accumulation. A fifth cluster with low/moderate levels of both modifications was also identified (C1). Importantly, these clusters positively and negatively correlated with transcription, respectively (Figure 6A). Although high H3K36me2 levels were linked to higher transcriptional activity, H3K27me3 enrichments were associated to low expression levels. Together, these data demonstrate that JMJD-5 regulate the homeostatic levels of independent mutually exclusive chromatin domains devoted to transcriptional activation (H3K36me2) and repression (H3K27me3).

The transcriptomic consequences of temperature shift have been studied (Belicard et al., 2018; Gómez-Orte et al., 2017; Ni et al., 2016), but the interplay between chromatin and temperature changes in *C. elegans* has been little explored (Costello and Petrella, 2019; Rogers and Phillips, 2020). Scatterplots comparing H3K36me2 and H3K27me3 levels in wild-type animals grown at 20°C and 25°C revealed that both marks increase with the enhancement of the temperature (Figure 6C, upper panels). However, when comparing H3K27 and H3K36 methylation level in *jmjd-5* mutants raised at 20°C and 25°C, we observed a slight temperature-dependent increase of H3K27me3 levels, but not a significant heat-induced H3K36me2 rise (Figure 6C, lower panels). The modulation of H3K36me2 and H3K27me3 with temperature in *jmjd-5* and wild-type animals can be better visualized when genes grouped by levels of these marks as shown in Figure 6A are considered (Figure 6D). Genes in the clusters showing the highest increased levels of H3K36me2 in *jmjd-5* mutant animals at 20°C (C3 and C5) were the same with the highest increase of this mark in wild-type animals at 25°C. Differently, genes in the clusters showing the highest increased levels of H3K27me3 in *jmjd-5* mutant animals at 20°C (C2 and C4) showed a further increase of H3K27me3 at high temperature (Figure 6D). All together, these evidences suggest that *jmjd-5* mutant animals can accommodate temperature-mediated changes in H3K27me3, but not the ones related to H3K36me2. Importantly, chromatin alterations observed in wild-type animals at high temperature were accompanied by a significative change of transcription in germ cells and when the germline transcriptomes of wild-type animals grown at the two temperatures were compared, about 700 DE genes ($p < 0.01$) were identified (Table S1), mainly grouped in categories relative to metabolism, ribosome biogenesis, and translation (Figure 6E). On the contrary, only 60 genes were DE ($p < 0.01$) when the germline transcriptomes of *jmjd-5* animals grown at 15°C and 25°C were compared (Table S1). These results therefore suggest that, in wild-type animals, an increase of temperature is accompanied by changes in chromatin landscape and in transcription in germ cells, and point to a deficiency of *jmjd-5* animals in this response associated to temperature change.

Loss of H3K27 demethylases partially restores *jmjd-5* fertility

To establish a strict connection between the aberrant epigenome landscape and fertility defects observed in *jmjd-5*, we further modulated the level of H3K36me2 and H3K27me3 by ablating the respective demethylases in *jmjd-5* mutant animals. Several demethylases are reported to remove H3K27me2/3, including members of the KDM6 (*utx-1*, *jmjd-3.1*, *jmjd-3.2*, *jmjd-3.3*) and KDM7 (*jmjd-1.2*) classes (Myers et al., 2018; Vandamme et al., 2012). Compound mutants were viable, and their fertility was assessed after seven generations at 25°C, when *jmjd-5(zr1234)* mutant animals are totally sterile. Removal of the catalytic activity of *utx-1* or ablation of single *jmjd-3* genes in *jmjd-5(zr1234)* genetic background did not have any effect on *jmjd-5(zr1234)* fertility (Figure S4A). However, the combined removal of *jmjd-3.1/2/3* genes in the *jmjd-5(zr1234)* genetic background (*jmjd-5;jmjd-3.1;jmjd-3.2;jmjd-3.3*) resulted in an

(C) Heatmaps of H3K27me3 ChIP-seq signal across H3K27me3 consensus peaks in N2 and *jmjd-5* mutants at 20°C and 25°C. Two replicates are shown.

(D) H3K27me3 scatterplots of 5-kb genome-wide bins signal. N2 versus *jmjd-5* at 20°C (left) and N2 versus *jmjd-5* at 25°C (right).

See also Figure S3.

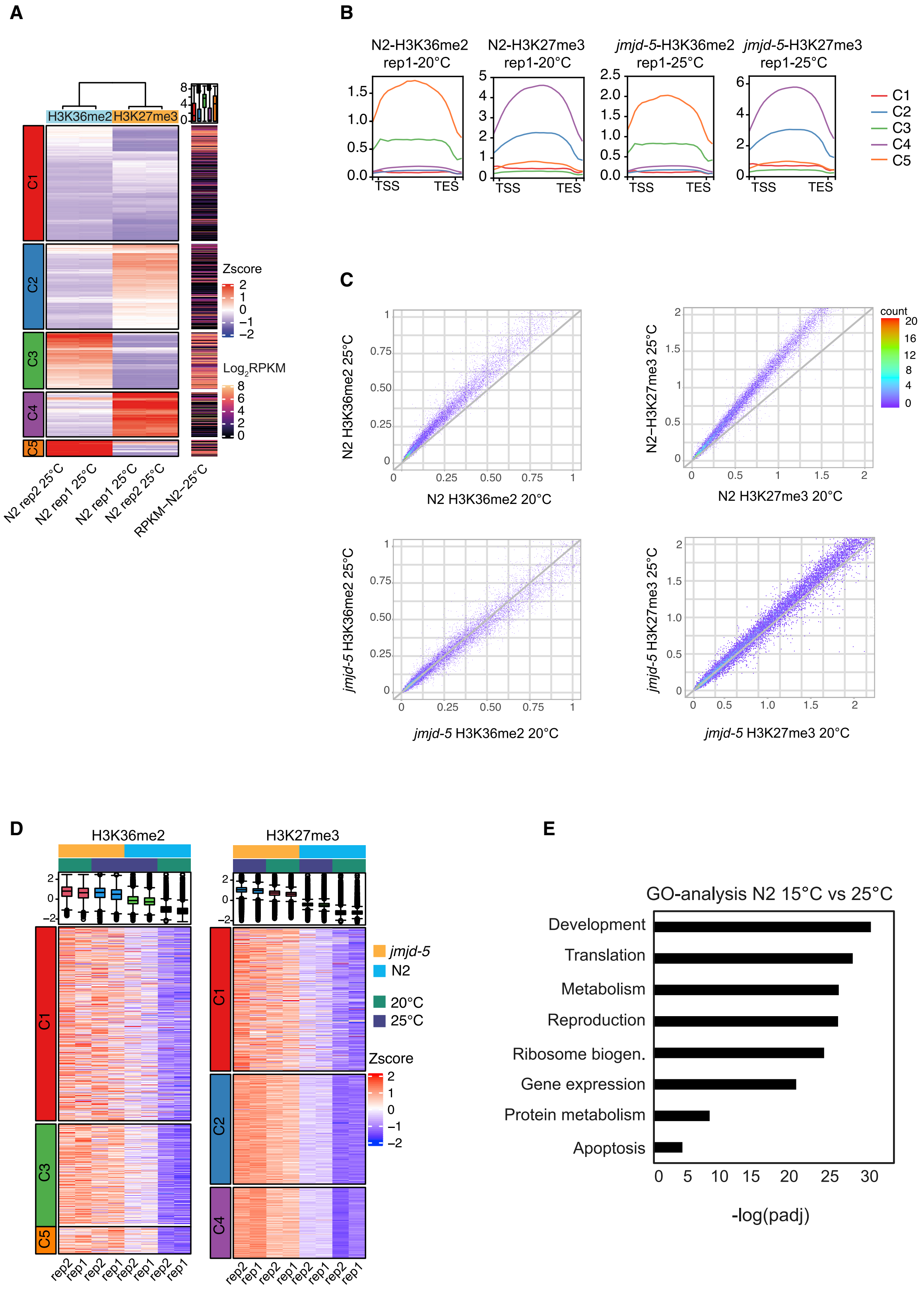

(legend on next page)

amelioration of the *jmjd-5(zr1234)* mortal germline phenotype at 25°C (Figure S4A). A similar rescuing effect was observed after the removal of *jmjd-1.2* (Figures S4A). Brood size measurement at different generations at 25°C confirmed that *jmjd-5;jmjd-3.1/2/3* and *jmjd-5;jmjd-1.2* mutant animals have sustained fertility for many generations at 25°C (Figure 7A). This amelioration was, however, partial and limited in time, and both compound mutants showed reduced brood size at generation 7, compared with wild-type, and became sterile after about 13 generations at 25°C (Figure 7A). Overall, these data suggest that preventing H3K27 methylation removal by specific demethylases counteracts, to a certain extent, the effects of loss of *jmjd-5* at high temperature. We concluded that increased level of H3K27me3 can play a compensatory effect that protects germline function when H3K36me2 is increased.

A similar approach was used to test the effect of loss of H3K36 demethylases in *jmjd-5* genetic background. *jmjd-2 and jhdm-1*, members of the KDM4 and KDM2 classes of H3K36 demethylases, respectively, are shown (Whetstine et al., 2006) or suggested, based on homology (Cheng, 2014; Tsukada et al., 2006), to remove methyl groups from H3K36. Despite showing a reduced brood size at 25°C, *jhdm-1(tm2828)* and *jmjd-2(tm2966)* mutants can be propagated at this temperature (Figure S4B), indicating the uniqueness of *jmjd-5* gene in preventing sterility at high temperature. Interestingly, *jmjd-5;jhdm1*, but not *jmjd-5;jmjd-2*, double-mutant animals showed a synergic effect on fertility, with an immediate sterility at 25°C (Figure S4B). Similarly, compound mutant animals lacking *jmjd-5* and *met-1*, a methyltransferase whose ablation results in decrease of H3K36me3 and in increased level of H3K36me2 (Cabianca et al., 2019) (Figure S4C) had a significantly reduced brood size at 20°C, compared with single mutants (Figure S4D), suggesting that a further increase of H3K36me2 is detrimental for fertility.

These results indicate that the temperature-sensitive mortal germline phenotype of *jmjd-5* is strictly connected to the aberrant level of H3K36 and H3K27 methylation, and that H3K36 and H3K27 demethylases provide an important contribution to germ cell immortality.

jmjd-5 and H3K27 demethylases cooperate to balance gene expression

To explore the transcriptional effects of H3K27 demethylases depletion in the *jmjd-5* genetic background, we compared the transcriptome of *jmjd-5* mutant animals obtained by RNA sequencing of whole adult animals grown at 25°C for three generations to the one from the *jmjd-5;jmjd-3.1/2/3* quadruple mutant, grown in the same conditions. DE genes ($p < 0.01$, Log2FC ± 1) identified in *jmjd-5*, *jmjd-3.1/2/3* triple mutant, and *jmjd-5;jmjd-3.1/2/3* quadruple mutant in comparison with wild-type are shown in Figure S5A and listed in Table S3. Using this approach, we identified 2,646 DE genes in *jmjd-5(zr1234)* mutants. Strikingly, the expression of a consistent number (1,667/2,646, 63%) of DE genes in *jmjd-5(zr1234)* was restored to wild-type level in *jmjd-5;jmjd-3.1/2/3* quadruple mutant (Figure 7B). A parallel approach was used to test the expression (Figure S5B) in *jmjd-1.2* and *jmjd-5; jmjd-1.2* double-mutant animals, and a similar trend was observed (Figure S5C). Comparison of the transcriptomes also revealed a high degree of overlap among DE genes identified in *jmjd-5(zr1234)* and in *jmjd-3.1/2/3* and *jmjd-1.2* (Figure 7C), which is particularly evident among upregulated genes, suggesting that these enzymes operate in a transcriptional framework and control, directly or indirectly, the expression of shared target genes. Despite the increased fertility at high temperature, the frail conditions of *jmjd-5;jmjd-3.1/2/3* quadruple mutant prevented the extraction of the germlines and the analysis of germ cells transcriptome. Although whole worm RNA sequencing has reduced sensitivity in detecting changes occurring in germ cells, we observed that a large percentage of the oogenic genes identified as DE in *jmjd-5* mutant animals whole worm RNA sequencing is restored to normal level in *jmjd-5;jmjd-3.1/2/3* (81/94) and in *jmjd-5;jmjd-1.2* (60/94). Altogether, these results strongly suggest that H3K36 and H3K27 demethylases cooperate to balance gene expression.

DISCUSSION

Recent investigations led to the identification of epigenetic-based mechanisms contributing to germline immortality; however, the role of histone demethylases remains largely elusive. Based on our data, we propose that JMJD-5 protects germ cell immortality at high temperature by preserving its naive state. In *jmjd-5* mutant animals, germ cell features are gradually compromised at high temperature, leading to loss of cell identity. These results support, at the organismal level, findings reported in human embryonic stem cells, in which loss of JMJD5/KDM8 causes reduced stemness and differentiation into multiple cell lineages (Zhu et al., 2014).

The homology of *jmjd-5* to JMJD5/KDM8, reported to demethylate H3K36me2 (Hsia et al., 2010; Ishimura et al., 2012; Marcon et al., 2014), together with the evidence of increased level of

Figure 6. Temperature effects on H3K36/K27 methylation and transcription

(A) Heatmap of H3K36me2 and H3K27me3 ChIP-seq signal on gene bodies enriched in H3K36me2 and/or H3K27me3 in N2 at 25°C. For each sample, ChIP-seq signal was transformed into *Z* scores to represent relative in-sample abundances of H3K36me2 and H3K27me3. K-means clustering (k = 5) was used to split H3K36me2$^+$/H3K27me3$^+$ genes in five different groups (C1–C5), annotated on the left of the heatmap. On the right, mRNA-seq expression −log2(RPKM+1) of the corresponding genes in N2 25°C samples. Data for two biological replicates are shown.

(B) Normalized H3K36me2 and H3K27me3 ChIP-seq signal across gene bodies in N2 and *jmjd-5* samples at 20°C and 25°C. One replica is shown.

(C) Scatterplots of H3K36me2 (left) and H3K27me3 (right) 5-kb genome-wide bins signal. N2 25°C versus N2 20°C (top) and *jmjd-5(zr1234)* 25°C versus *jmjd-5(zr1234)* 20°C (bottom) are shown.

(D) Heatmaps of H3K36me2 and H3K27me3 ChIP-seq signal on gene bodies from clusters identified in (A). For each histone modification, clusters with low, medium, and high enrichments are shown.

(E) GO analysis of DE genes ($p < 0.01$) identified by RNA sequencing of isolated germlines of N2 grown at 15°C or at 25°C for three generations (F3). Selected GO terms are presented as $-\log_{10}$(p-adjusted).

TES, transcription end site; TSS, transcription start site.

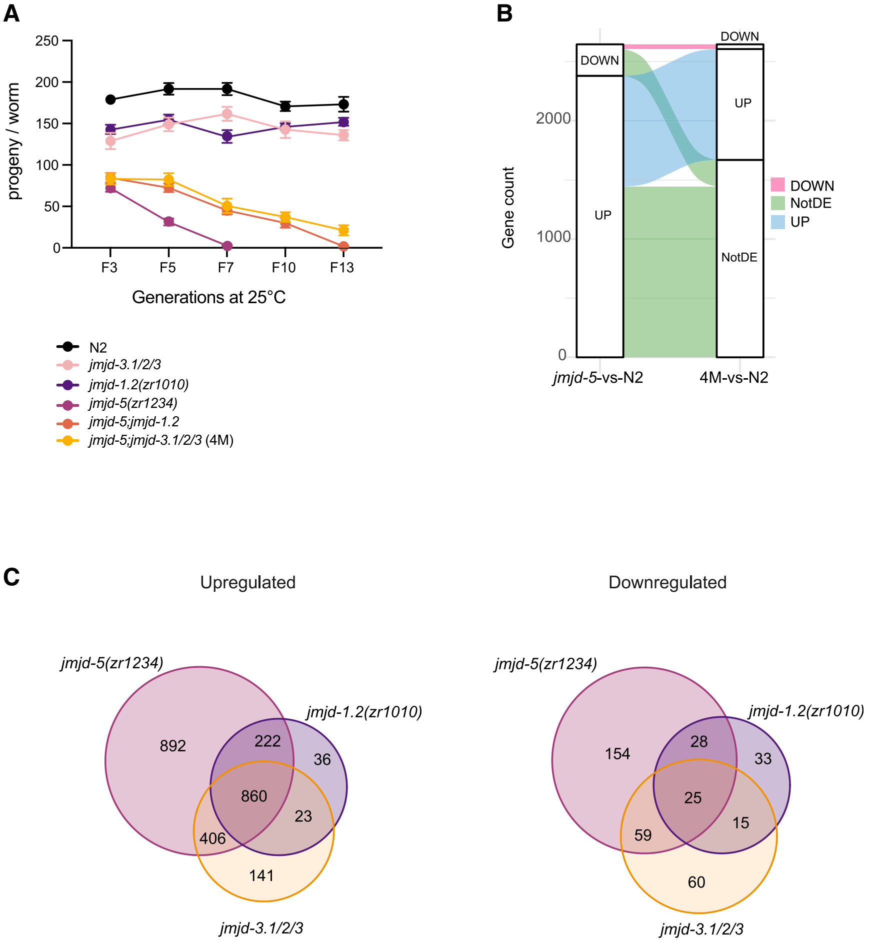

Figure 7. Loss of H3K27me2/3 demethylases ameliorates *jmjd-5* defects

(A) Brood size of indicated strains grown at 25°C, at the indicate generations. Bars represent SEM; n > 15.

(B) Alluvial diagram of differentially expressed genes from *jmjd-5* versus N2 (25°C) and 4M versus N2 (25°C). 4M indicates *jmjd-5;jmjd-3.1/2/3* quadruple mutant.

(C) Venn diagrams showing the overlap of upregulated (left) and downregulated (right) genes (p-adjusted < 0.01 and Log2FC ± 1) identified in indicated strains grown at 25°C for three generations (F3).

See also Figures S4 and S5.

H3K36me2 (Amendola et al., 2017; this study), strongly suggests that *jmjd-5* is a H3K36me2 demethylase in *C. elegans*. *jmjd-5* is apparently not required to constrain H3K36me2 regions or to remove spurious H3K36me2 deposition, but rather to prevent an accumulation of H3K36me2 in regions that are normally marked by this modification. Intriguingly, the increase of H3K36me2 correlates with a rise of H3K27me3 levels in H3K27me3-positive regions. This unexpected result might suggest that JMJD-5, similar to other histone demethylases (Myers et al., 2018; Whetstine et al., 2006), might have multiple specificity and act also as a demethylase for H3K27me3. Biochemical approaches are needed to investigate this possibility. However, the rescuing effect observed when H3K27 demethylases are ablated in *jmjd-5* background suggests that the H3K27me3 rise in *jmjd-5* mutant is not directly mediated by JMJD-5 catalytic activity abrogation, but rather the result of a protective and secondary event that takes place to balance the aberrant increase of H3K36me2. It is worth noting that a similar H3K27 methylation increase is observed in germ cells after depletion of *set-2*, the major methyltransferase for H3K4 (Robert et al., 2014), suggesting that the modulation of H3K27 methylation might act as a general compensatory mechanism when the chromatin landscape of germ cells is compromised. The evidence that *jmjd-5* mutant animals can still accommodate changes in H3K27me3 level, even if at a lower extent than wild-type animals, but not of H3K36me2 at high temperature, also reinforces the notion that the increased levels of H3K36 and H3K27 methylation are mediated by two correlated but distinct enzymatic machineries. Possible explanations for this effect on H3K27 methylation could be linked to a persistent activity of the PRC2 complex in H3K27me3-positive regions, where it is already recruited, or to the involvement of another, yet unknown, H3K27 methyltransferase (Bender et al., 2004), or to a direct role of H3K27 demethylases.

Despite the increase of H3K36/K27 methylation at 20°C, *jmjd-5* mutant animals are fertile in this condition. This evidence suggests that in *jmjd-5* mutant animals the levels of H3K36/K27 methylation reach an equilibrium that ensures germ cell functionality at 20°C, and that this balance is challenged at high temperature. Indeed, we observed a coordinate increment in H3K36 and H3K27 methylation level in wild-type animals when exposed to high temperatures. Although the rise in H3K27me3 is still evident, the increase of H3K36me2 is abolished or hidden in *jmjd-5* mutant animals, indicating that *jmjd-5* contributes to the physiological response to the temperature increase and to the adaptation of the animals to new conditions. Thus, we hypothesize that *jmjd-5* animals at 20°C have a H3K36me2 pattern that resembles the one of wild-type at high temperature and reaches a "plateau" state for H3K36me2, disabling the worm to further sustain the temperature-mediated H3K36me2 increase. The evidence that removal of H3K27 or H3K36 demethylases alleviates or worsens the *jmjd-5* temperature-sensitive fertility defect suggests that these demethylases contribute to the H3K36/K27 cross-talk and have the potential to buffer it. Therefore, based on the coordinate modulation of H3K36/K27 methylation in *jmjd-5* mutants and on the transcriptome of *jmjd-5*, *jmjd-1.2*, *jmjd-3.1/2/3*, and compound mutants, we postulate the existence of a H3K36/K27 demethylase-based system that controls and fine-tunes the H3K36/K27 cross-talk

and transcription. This system parallels, and possibly integrates, the H3K36/K27 methyltransferase-based system made by *mes-2* and *mes-4*, with a major difference: although *mes-2* and *mes-4* simultaneous removal results in a larger number of deregulated genes and in a worsening of the phenotype (Gaydos et al., 2012), compound mutants of *jmjd-5* and *jmjd-3.1/2/3* and *jmjd-1.2* have a restored transcription of target genes and an improved fertility at high temperature. It should be noted that, when the whole-animal transcriptome was analyzed, ablation of *jmjd-5*, *jmjd-3 s*, and *jmjd-1,2* resulted mainly in gene upregulation. Although this effect is consistent with an increase of H3K36 methylation, it is surprising in animals accumulating methylated H3K27, a well-established repressive post-translational modification. Even more unexpected is the rescuing effect observed in *jmjd-5*, *jmjd-3 s*, and *jmjd-1.2* compound mutant animals at the transcription level. Although the identification of direct targets of these demethylases is required to solve these incongruities, it is interesting to note that, in agreement with our results, overexpression of *jmjd-1.2* and *jmjd-3.1* resulted in a consistent, yet unexpected, downregulation of commonly regulated genes (Merkwirth et al., 2016).

The coordinate increment in H3K36 and H3K27 methylation level observed in wild-type animals when exposed to high temperatures suggests that the landscape of histone modifications might change in response to external conditions to better accommodate adjustments in metabolism and cellular activities required in the new environmental settings. Although multiple studies in plants (He et al., 2021) indicate that chromatin can be affected by temperature variation, little is known in other organisms. Recent works in *C. elegans* describe changes in chromatin compaction in wild-type animals raised at high temperature (Costello and Petrella, 2019; Rogers and Phillips, 2020) and support the possibility that chromatin conformation might be susceptible to temperature changes.

The progressive nature of *jmjd-5* phenotypes over generations remains at the moment unclear, and we can envision two, not mutually exclusive, possible explanations. First, the evidence that in *jmjd-5* mutant animals P-granules decrease over generations might suggest a contribution of *hrde-1*-independent small RNAs pathways, because transient changes in P-granules have been shown to affect the pools of small RNAs with transgenerational consequences (Dodson and Kennedy, 2019; Lev and Rechavi, 2020; Lev et al., 2019). Second, as H3K36 methylation marks genes expressed in germline whose memory need to be transmitted to the next generation, the increased level of H3K36 methylation could result in a "bad memory" of genes that are expressed in *jmjd-5* mutant germlines.

Overall, our study provides further evidence that a precise balance of H3K36 and H3K27 methylation level is strictly required for germline functions and emphasizes the role of H3K36/K27 histone demethylases in maintaining this equilibrium, pointing to their relevant contribution in the adaptation to environmental condition changes.

Limitation of the study

The results of ChIP, performed in whole animals (where the germ cells account for about 50% of the material), cannot be strictly connected to the transcriptome profile of germ cells. Because we observed an increase of H3K27/36 methylation in *jmjd-5* germ cells (Amendola et al., 2017) (this study), we assume that the ChIP signals are, at least in part, derived by germ cells. Further analyses using chromatin extracted from germlines will provide a more direct link between temperature shift, chromatin landscape, and transcription in germ cell changes.

STAR★METHODS

Detailed methods are provided in the online version of this paper and include the following:

- KEY RESOURCES TABLE
- RESOURCE AVAILABILITY
  - Lead contact
  - Materials availability
  - Data and code availability
- EXPERIMENTAL MODEL AND SUBJECT DETAILS
  - Animals
- METHOD DETAILS
  - Brood size
  - Mortal germline assay
  - Endomitotic eggs and germline length
  - Reversibility assay
  - Trans-differentiation assay
  - Germ cell apoptosis assay
  - Immunofluorescence
  - IF Imaging and quantification
  - Germline RNA sequencing
  - Classification of DE genes in categories
  - X chromosome to Autosome ratio
  - DE genes comparison to other studies
  - Whole worm RNA sequencing
  - Chromatin immunoprecipitation (ChIP)
  - Bioinformatic analyses
- QUANTIFICATION AND STATISTICAL ANALYSIS

SUPPLEMENTAL INFORMATION

Supplemental information can be found online at https://doi.org/10.1016/j.celrep.2021.110050.

ACKNOWLEDGMENTS

The authors thank Shruti Pande, Line Kerimback Kikarsen, and Yasuko Antoku for technical help. Some strains used are from the *Caenorhabditis* Genetics Center (CGC), funded by NIH Office of Research Infrastructure Programs (P40 OD010440), and the National BioResource project for C. elegans (Japan). This work was supported by the Danish National Research Foundation (DNRF82 to A.E.S.). The work of F.R., D.F.P., and D.P. was supported by the Italian Association for Cancer Research (IG-2017-20290) and the European Research Council (EC-H2020-ERC-CoG-DissectPcG: 725268). D.F.P. was supported by a FIRC fellowship.

AUTHOR CONTRIBUTIONS

N.Z., K.M., F.R., D.F.P., S.D., U.P., and P.G.A. carried out the experimental work and analyzed the data. A.E.S., D.P., K.K., N.Z., and K.M. designed the experiments. A.E.S directed the study. A.E.S. wrote the manuscript with the help of D.P.

DECLARATION OF INTERESTS

The authors declare no competing interests.

Received: May 21, 2021
Revised: September 18, 2021
Accepted: November 2, 2021
Published: November 23, 2021

REFERENCES

Ahringer, J., and Gasser, S.M. (2018). Repressive Chromatin in *Caenorhabditis elegans*: Establishment, Composition, and Function. Genetics *208*, 491–511.

Amendola, P.G., Zaghet, N., Ramalho, J.J., Vilstrup Johansen, J., Boxem, M., and Salcini, A.E. (2017). JMJD-5/KDM8 regulates H3K36me2 and is required for late steps of homologous recombination and genome integrity. PLoS Genet. *13*, e1006632.

Belicard, T., Jareosettasin, P., and Sarkies, P. (2018). The piRNA pathway responds to environmental signals to establish intergenerational adaptation to stress. BMC Biol. *16*, 103.

Bender, L.B., Cao, R., Zhang, Y., and Strome, S. (2004). The MES-2/MES-3/MES-6 complex and regulation of histone H3 methylation in C. elegans. Curr. Biol. *14*, 1639–1643.

Bender, L.B., Suh, J., Carroll, C.R., Fong, Y., Fingerman, I.M., Briggs, S.D., Cao, R., Zhang, Y., Reinke, V., and Strome, S. (2006). MES-4: an autosome-associated histone methyltransferase that participates in silencing the X chromosomes in the C. elegans germ line. Development *133*, 3907–3917.

Benjamini, Y.H. (1995). Controlling the false discovery rate: a practical and powerful approach to multiple testing. J. R. Stat. Soc. B Stat. Methodol. *57*, 289–300.

Bolger, A.M., Lohse, M., and Usadel, B. (2014). Trimmomatic: a flexible trimmer for Illumina sequence data. Bioinformatics *30*, 2114–2120.

Brangwynne, C.P., Eckmann, C.R., Courson, D.S., Rybarska, A., Hoege, C., Gharakhani, J., Jülicher, F., and Hyman, A.A. (2009). Germline P granules are liquid droplets that localize by controlled dissolution/condensation. Science *324*, 1729–1732.

Brenner, S. (1974). The genetics of Caenorhabditis elegans. Genetics *77*, 71–94.

Buckley, B.A., Burkhart, K.B., Gu, S.G., Spracklin, G., Kershner, A., Fritz, H., Kimble, J., Fire, A., and Kennedy, S. (2012). A nuclear Argonaute promotes multigenerational epigenetic inheritance and germline immortality. Nature *489*, 447–451.

Cabianca, D.S., Muñoz-Jiménez, C., Kalck, V., Gaidatzis, D., Padeken, J., Seeber, A., Askjaer, P., and Gasser, S.M. (2019). Active chromatin marks drive spatial sequestration of heterochromatin in C. elegans nuclei. Nature *569*, 734–739.

Cheng, X. (2014). Structural and functional coordination of DNA and histone methylation. Cold Spring Harb. Perspect. Biol. *6*, a018747.

Costello, M.E., and Petrella, L.N. (2019). *C. elegans* synMuv B proteins regulate spatial and temporal chromatin compaction during development. Development *146*, dev174383.

Dobin, A., and Gingeras, T.R. (2015). Mapping RNA-seq Reads with STAR. Curr. Protoc. Bioinformatics *51*, 11.14.1–11.14.19.

Dodson, A.E., and Kennedy, S. (2019). Germ Granules Coordinate RNA-Based Epigenetic Inheritance Pathways. Dev. Cell *50*, 704–715.e4.

Evans, K.J., Huang, N., Stempor, P., Chesney, M.A., Down, T.A., and Ahringer, J. (2016). Stable *Caenorhabditis elegans* chromatin domains separate broadly expressed and developmentally regulated genes. Proc. Natl. Acad. Sci. USA *113*, E7020–E7029.

Faust, G.G., and Hall, I.M. (2014). SAMBLASTER: fast duplicate marking and structural variant read extraction. Bioinformatics *30*, 2503–2505.

Finogenova, K., Bonnet, J., Poepsel, S., Schäfer, I.B., Finkl, K., Schmid, K., Litz, C., Strauss, M., Benda, C., and Müller, J. (2020). Structural basis for PRC2 decoding of active histone methylation marks H3K36me2/3. eLife *9*, e61964.

Fong, Y., Bender, L., Wang, W., and Strome, S. (2002). Regulation of the different chromatin states of autosomes and X chromosomes in the germ line of C. elegans. Science *296*, 2235–2238.

Furuhashi, H., and Kelly, W.G. (2010). The epigenetics of germ-line immortality: lessons from an elegant model system. Dev. Growth Differ. *52*, 527–532.

Furuhashi, H., Takasaki, T., Rechtsteiner, A., Li, T., Kimura, H., Checchi, P.M., Strome, S., and Kelly, W.G. (2010). Trans-generational epigenetic regulation of C. elegans primordial germ cells. Epigenetics Chromatin *3*, 15.

Gaydos, L.J., Rechtsteiner, A., Egelhofer, T.A., Carroll, C.R., and Strome, S. (2012). Antagonism between MES-4 and Polycomb repressive complex 2 promotes appropriate gene expression in C. elegans germ cells. Cell Rep. *2*, 1169–1177.

Gómez-Orte, E., Cornes, E., Zheleva, A., Sáenz-Narciso, B., de Toro, M., Iñiguez, M., López, R., San-Juan, J.F., Ezcurra, B., Sacristán, B., et al. (2017). Effect of the diet type and temperature on the *C. elegans* transcriptome. Oncotarget *9*, 9556–9571.

He, K., Cao, X., and Deng, X. (2021). Histone methylation in epigenetic regulation and temperature responses. Curr. Opin. Plant Biol. *61*, 102001.

Hsia, D.A., Tepper, C.G., Pochampalli, M.R., Hsia, E.Y., Izumiya, C., Huerta, S.B., Wright, M.E., Chen, H.W., Kung, H.J., and Izumiya, Y. (2010). KDM8, a H3K36me2 histone demethylase that acts in the cyclin A1 coding region to regulate cancer cell proliferation. Proc. Natl. Acad. Sci. USA *107*, 9671–9676.

Ignatiadis, N., Klaus, B., Zaugg, J.B., and Huber, W. (2016). Data-driven hypothesis weighting increases detection power in genome-scale multiple testing. Nat. Methods *13*, 577–580.

Ishimura, A., Minehata, K., Terashima, M., Kondoh, G., Hara, T., and Suzuki, T. (2012). Jmjd5, an H3K36me2 histone demethylase, modulates embryonic cell proliferation through the regulation of Cdkn1a expression. Development *139*, 749–759.

Katz, D.J., Edwards, T.M., Reinke, V., and Kelly, W.G. (2009). A C. elegans LSD1 demethylase contributes to germline immortality by reprogramming epigenetic memory. Cell *137*, 308–320.

Kim, D., Pertea, G., Trapnell, C., Pimentel, H., Kelley, R., and Salzberg, S.L. (2013). TopHat2: accurate alignment of transcriptomes in the presence of insertions, deletions and gene fusions. Genome Biol. *14*, R36.

Knutson, A.K., Egelhofer, T., Rechtsteiner, A., and Strome, S. (2017). Germ Granules Prevent Accumulation of Somatic Transcripts in the Adult *Caenorhabditis elegans* Germline. Genetics *206*, 163–178.

Langmead, B. (2010). Aligning short sequencing reads with Bowtie. Curr. Protoc. Bioinformatics *32*, 11.7.1–11.7.14.

Lev, I., and Rechavi, O. (2020). Germ Granules Allow Transmission of Small RNA-Based Parental Responses in the "Germ Plasm". iScience *23*, 101831.

Lev, I., Toker, I.A., Mor, Y., Nitzan, A., Weintraub, G., Antonova, O., Bhonkar, O., Ben Shushan, I., Seroussi, U., Claycomb, J.M., et al. (2019). Germ Granules Govern Small RNA Inheritance. Curr. Biol. *29*, 2880–2891.e4.

Liao, Y., Smyth, G.K., and Shi, W. (2014). featureCounts: an efficient general purpose program for assigning sequence reads to genomic features. Bioinformatics *30*, 923–930.

Love, M.I., Huber, W., and Anders, S. (2014). Moderated estimation of fold change and dispersion for RNA-seq data with DESeq2. Genome Biol. *15*, 550.

Marcon, E., Ni, Z., Pu, S., Turinsky, A.L., Trimble, S.S., Olsen, J.B., Silverman-Gavrila, R., Silverman-Gavrila, L., Phanse, S., Guo, H., et al. (2014). Human-chromatin-related protein interactions identify a demethylase complex required for chromosome segregation. Cell Rep. *8*, 297–310.

Marnik, E.A., Fuqua, J.H., Sharp, C.S., Rochester, J.D., Xu, E.L., Holbrook, S.E., and Updike, D.L. (2019). Germline Maintenance Through the Multifaceted Activities of GLH/Vasa in *Caenorhabditis elegans* P Granules. Genetics *213*, 923–939.

McMurchy, A.N., Stempor, P., Gaarenstroom, T., Wysolmerski, B., Dong, Y., Aussianikava, D., Appert, A., Huang, N., Kolasinska-Zwierz, P., Sapetschnig,

A., et al. (2017). A team of heterochromatin factors collaborates with small RNA pathways to combat repetitive elements and germline stress. eLife *6*, e21666.

Merkwirth, C., Jovaisaite, V., Durieux, J., Matilainen, O., Jordan, S.D., Quiros, P.M., Steffen, K.K., Williams, E.G., Mouchiroud, L., Tronnes, S.U., et al. (2016). Two Conserved Histone Demethylases Regulate Mitochondrial Stress-Induced Longevity. Cell *165*, 1209–1223.

Myers, T.R., Amendola, P.G., Lussi, Y.C., and Salcini, A.E. (2018). JMJD-1.2 controls multiple histone post-translational modifications in germ cells and protects the genome from replication stress. Sci. Rep. *8*, 3765.

Ni, J.Z., Chen, E., and Gu, S.G. (2014). Complex coding of endogenous siRNA, transcriptional silencing and H3K9 methylation on native targets of germline nuclear RNAi in C. elegans. BMC Genomics *15*, 1157.

Ni, J.Z., Kalinava, N., Chen, E., Huang, A., Trinh, T., and Gu, S.G. (2016). A transgenerational role of the germline nuclear RNAi pathway in repressing heat stress-induced transcriptional activation in C. elegans. Epigenetics Chromatin *9*, 3.

Orlando, D.A., Chen, M.W., Brown, V.E., Solanki, S., Choi, Y.J., Olson, E.R., Fritz, C.C., Bradner, J.E., and Guenther, M.G. (2014). Quantitative ChIP-Seq normalization reveals global modulation of the epigenome. Cell Rep. *9*, 1163–1170.

Ortiz, M.A., Noble, D., Sorokin, E.P., and Kimble, J. (2014). A new dataset of spermatogenic vs. oogenic transcriptomes in the nematode Caenorhabditis elegans. G3 (Bethesda) *4*, 1765–1772.

Padeken, J., Methot, S., Zeller, P., Delaney, C.E., Kalck, V., and Gasser, S.M. (2021). Argonaute NRDE-3 and MBT domain protein LIN-61 redundantly recruit an H3K9me3 HMT to prevent embryonic lethality and transposon expression. Genes Dev. *35*, 82–101.

Patel, T., Tursun, B., Rahe, D.P., and Hobert, O. (2012). Removal of Polycomb repressive complex 2 makes C. elegans germ cells susceptible to direct conversion into specific somatic cell types. Cell Rep. *2*, 1178–1186.

Ramírez, F., Ryan, D.P., Grüning, B., Bhardwaj, V., Kilpert, F., Richter, A.S., Heyne, S., Dündar, F., and Manke, T. (2016). deepTools2: a next generation web server for deep-sequencing data analysis. Nucleic Acids Res. *44* (*W1*), W160–W165.

Rechtsteiner, A., Ercan, S., Takasaki, T., Phippen, T.M., Egelhofer, T.A., Wang, W., Kimura, H., Lieb, J.D., and Strome, S. (2010). The histone H3K36 methyltransferase MES-4 acts epigenetically to transmit the memory of germline gene expression to progeny. PLoS Genet. *6*, e1001091.

Reed, K.J., Svendsen, J.M., Brown, K.C., Montgomery, B.E., Marks, T.N., Vijayasarathy, T., Parker, D.M., Nishimura, E.O., Updike, D.L., and Montgomery, T.A. (2020). Widespread roles for piRNAs and WAGO-class siRNAs in shaping the germline transcriptome of Caenorhabditis elegans. Nucleic Acids Res. *48*, 1811–1827.

Robert, V.J., Mercier, M.G., Bedet, C., Janczarski, S., Merlet, J., Garvis, S., Ciosk, R., and Palladino, F. (2014). The SET-2/SET1 histone H3K4 methyltransferase maintains pluripotency in the Caenorhabditis elegans germline. Cell Rep. *9*, 443–450.

Robert, V.J., Knutson, A.K., Rechtsteiner, A., Garvis, S., Yvert, G., Strome, S., and Palladino, F. (2020). *Caenorhabditis elegans* SET1/COMPASS Maintains Germline Identity by Preventing Transcriptional Deregulation Across Generations. Front. Cell Dev. Biol. *8*, 561791.

Rogers, A.K., and Phillips, C.M. (2020). RNAi pathways repress reprogramming of C. elegans germ cells during heat stress. Nucleic Acids Res. *48*, 4256–4273.

Seelk, S., Adrian-Kalchhauser, I., Hargitai, B., Hajduskova, M., Gutnik, S., Tursun, B., and Ciosk, R. (2016). Increasing Notch signaling antagonizes PRC2-mediated silencing to promote reprograming of germ cells into neurons. eLife *5*, e15477.

Seydoux, G. (2018). The P Granules of C. elegans: A Genetic Model for the Study of RNA-Protein Condensates. J. Mol. Biol. *430*, 4702–4710.

Sheth, U., Pitt, J., Dennis, S., and Priess, J.R. (2010). Perinuclear P granules are the principal sites of mRNA export in adult C. elegans germ cells. Development *137*, 1305–1314.

Smelick, C., and Ahmed, S. (2005). Achieving immortality in the C. elegans germline. Ageing Res. Rev. *4*, 67–82.

Spichal, M., Heestand, B., Billmyre, K.K., Frenk, S., Mello, C.C., and Ahmed, S. (2021). Germ granule dysfunction is a hallmark and mirror of Piwi mutant sterility. Nat. Commun. *12*, 1420.

Streubel, G., Watson, A., Jammula, S.G., Scelfo, A., Fitzpatrick, D.J., Oliviero, G., McCole, R., Conway, E., Glancy, E., Negri, G.L., et al. (2018). The H3K36me2 Methyltransferase Nsd1 Demarcates PRC2-Mediated H3K27me2 and H3K27me3 Domains in Embryonic Stem Cells. Mol. Cell *70*, 371–379.e5.

Strome, S., and Wood, W.B. (1983). Generation of asymmetry and segregation of germ-line granules in early C. elegans embryos. Cell *35*, 15–25.

Strome, S., Kelly, W.G., Ercan, S., and Lieb, J.D. (2014). Regulation of the X chromosomes in Caenorhabditis elegans. Cold Spring Harb. Perspect. Biol. *6*, a018366.

Tabuchi, T.M., Rechtsteiner, A., Strome, S., and Hagstrom, K.A. (2014). Opposing activities of DRM and MES-4 tune gene expression and X-chromosome repression in Caenorhabditis elegans germ cells. G3 (Bethesda) *4*, 143–153.

Tsukada, Y., Fang, J., Erdjument-Bromage, H., Warren, M.E., Borchers, C.H., Tempst, P., and Zhang, Y. (2006). Histone demethylation by a family of JmjC domain-containing proteins. Nature *439*, 811–816.

Tursun, B., Patel, T., Kratsios, P., and Hobert, O. (2011). Direct conversion of C. elegans germ cells into specific neuron types. Science *331*, 304–308.

Updike, D., and Strome, S. (2010). P granule assembly and function in Caenorhabditis elegans germ cells. J. Androl. *31*, 53–60.

Updike, D.L., Knutson, A.K., Egelhofer, T.A., Campbell, A.C., and Strome, S. (2014). Germ-granule components prevent somatic development in the C. elegans germline. Curr. Biol. *24*, 970–975.

Vandamme, J., Lettier, G., Sidoli, S., Di Schiavi, E., Nørregaard Jensen, O., and Salcini, A.E. (2012). The C. elegans H3K27 demethylase UTX-1 is essential for normal development, independent of its enzymatic activity. PLoS Genet. *8*, e1002647.

Wang, X., Zhao, Y., Wong, K., Ehlers, P., Kohara, Y., Jones, S.J., Marra, M.A., Holt, R.A., Moerman, D.G., and Hansen, D. (2009). Identification of genes expressed in the hermaphrodite germ line of C. elegans using SAGE. BMC Genomics *10*, 213.

Weiser, N.E., and Kim, J.K. (2019). Multigenerational Regulation of the *Caenorhabditis elegans* Chromatin Landscape by Germline Small RNAs. Annu. Rev. Genet. *53*, 289–311.

Whetstine, J.R., Nottke, A., Lan, F., Huarte, M., Smolikov, S., Chen, Z., Spooner, E., Li, E., Zhang, G., Colaiacovo, M., and Shi, Y. (2006). Reversal of histone lysine trimethylation by the JMJD2 family of histone demethylases. Cell *125*, 467–481.

Yu, G., Wang, L.G., and He, Q.Y. (2015). ChIPseeker: an R/Bioconductor package for ChIP peak annotation, comparison and visualization. Bioinformatics *31*, 2382–2383.

Zeller, P., Padeken, J., van Schendel, R., Kalck, V., Tijsterman, M., and Gasser, S.M. (2016). Histone H3K9 methylation is dispensable for Caenorhabditis elegans development but suppresses RNA:DNA hybrid-associated repeat instability. Nat. Genet. *48*, 1385–1395.

Zhang, Y., Liu, T., Meyer, C.A., Eeckhoute, J., Johnson, D.S., Bernstein, B.E., Nusbaum, C., Myers, R.M., Brown, M., Li, W., and Liu, X.S. (2008). Model-based analysis of ChIP-Seq (MACS). Genome Biol. *9*, R137.

Zhu, H., Hu, S., and Baker, J. (2014). JMJD5 regulates cell cycle and pluripotency in human embryonic stem cells. Stem Cells *32*, 2098–2110.

Zhu, A., Ibrahim, J.G., and Love, M.I. (2019). Heavy-tailed prior distributions for sequence count data: removing the noise and preserving large differences. Bioinformatics *35*, 2084–2092.

STAR★METHODS

KEY RESOURCES TABLE

REAGENT or RESOURCE	SOURCE	IDENTIFIER
Antibodies		
Anti-H3K27me3	Millipore	Cat#07-449;RRID:AB_310624
Anti-H3K27me3	Wako/MAB Institute Inc.	Cat#MABI0323;RRID:AB_11123929
Anti-H3K36me2	abcam	Cat#Ab9049;RRID:AB_1280939
Anti-H3K36me3	Active motif	Cat#61902;RRID:AB_2615073
Anti-PGL-1	DSHB	Cat#K76;RRID:AB_531836
Alexa fluor 568 anti-rabbit	Life technologies	Cat#A11036;RRID:AB_10563566
Bacterial strains		
E. coli OP50	Authors' lab	OP50
Chemical, peptides, and recombinant proteins		
Trizol reagent	Thermo Scientific	***Cat#15596026***
Protease inhibitor cocktail tablets	Roche	Cat#11873580001
Amersham Protran	GE healthcare	Cat#10600003
Triton X-100	Sigma Aldrich	Cat#T8787
DAPI	Sigma Aldrich	Cat#D9542
Formaldehyde	Sigma Aldrich	Cat#252549
Protein-G Dynabeads	Invitrogen	Cat#10004D
Critical commercial assays		
NEBNext Ultra II DNA Library Prep Kit for Illumina	New England BioLabs	Cat#E7645S
NEBNext multiplex Oligos for Illumina	New England BioLabs	Cat#E7335S, E7500S
TruSeq RNA Library Prep Kit v2	Illumina	Cat#RS-122-2001
NextSeq 500/550 High Output Kit v2	Illumina	Cat#FC-404-2005
RNase-Free DNase Set	QIAGEN	Cat#79254
SuperSignal West Pico PLUS Chemiluminescent Substrate	Thermo Scientific	Cat#34577
Immobilon Western Chemiluminescent HRP Substrate	Millipore	Cat#WBKLS0500
KAPA taq ReadyMix	Sigma Aldrich	Cat#KK1006
PCR MinElute PCR purification kit	QIAGEN	Cat#28006
Deposited data		
Sequencing data (ChIP and RNA)	This study	GEO: GSE174652
Experimental models: Organisms/strains (*C. elegans*)		
wild-type, Bristol	CGC	N2
jmjd-5(tm3735) V	NBRP	N/A
jmjd-5(zr1234) V	(Amendola et al., 2017)	N/A
jmjd-3.1(gk384) X	CGC	VC936
jmjd-3.2(tm3121) X	NBRP	N/A
jmjd-3.3(tm3197) X	NBRP	N/A
jmjd-5(syb1946) V	This study	PHX1946
utx-1(tm3118)+UTX-1DD::GFP, rol-6 X	(Vandamme et al., 2012)	N/A
jmjd-1.2(zr1010) IV	(Myers et al., 2018)	N/A
lin-17(n671)I; unc-119(e2498) III; him-5(e1490) V; mhIs9(lin-17::GFP)	CGC	KS411
unc-119(e2498) III	CGC	CB4845
him-5(e1490) V	CGC	CB4088

(*Continued on next page*)

Continued

REAGENT or RESOURCE	SOURCE	IDENTIFIER
edIs6 [unc-119::GFP + rol-6(su1006)] IV	CGC	DP132
otEx304[unc-75p::GFP+ rol6(su1006)]	CGC	OH443
hrde-1(tm1200) III	CGC	YY538
cep-1(gk138) I	CGC	VC172
jhdm-1(tm2828) III	NBRP	T26A5.5
jmjd-2(tm2966) II	NBRP	Y48B6A.11
met-1(n4337) I	CGC	MT16973
met-2(n4256) III	CGC	RB1789
jmjd-5(zr1234);jhdm-1(tm2828)	This study	N/A
jmjd-5(zr1234);met-1(n4337)	This study	N/A
jmjd-5 (zr1234);met-2(n4256)	This study	N/A
jmjd-5(zr1234);jmjd-1.2(zr1010)	This study	N/A
jmjd-5(zr1234);utx-1(tm3118)+UTX-1DD::GFP	This study	N/A
jmjd-5(zr1234);jmjd-3.1 (gk384)	This study	N/A
jmjd-5(zr1234);jmjd-3.2(tm3121)	This study	N/A
jmjd-5(zr1234);jmjd-3.3(tm3197)	This study	N/A
jmjd-5(zr1234);jmjd-3.1-3.2-3.3	This study	N/A
jmjd-5(tm3735);cep-1(gk138)	This study	N/A
jmjd-5(zr1234);hrde-1(tm1200)	This study	N/A
*jmjd-5(zr1234);*mhIs9[lin-17::GFP]	This study	N/A
jmjd-5(zr1234); edIs6 [unc-119::GFP + rol-6(su1006)]	This study	N/A
jmjd-5(zr1234); otEx304[unc-75p::GFP + rol6(su1006)]	This study	N/A
Software and algorithms		
GraphPad Prism 9	GraphPadSoftware, La Jolla, CA	https://www.graphpad.com/scientific-software/prism/
ImageJ	NIH image	https://imagej.nih.gov/ij/
Bowtie1	(Langmead, 2010)	http://bowtie-bio.sourceforge.net/manual.shtml
Samblaster	(Faust and Hall, 2014)	https://github.com/GregoryFaust/samblaster
MACS2	(Zhang et al., 2008)	https://github.com/taoliu/MACS
ChIPseeker	(Yu et al., 2015)	https://bioconductor.org/packages/release/bioc/html/ChIPseeker.html
DeepTools	(Ramírez et al., 2016)	https://deeptools.readthedocs.io/en/develop/
STAR	(Dobin and Gingeras, 2015)	N/A
FeatureCounts	(Liao et al., 2014)	http://subread.sourceforge.net/
DESeq2	(Love et al., 2014)	https://bioconductor.org/packages/release/bioc/html/DESeq2.html
Ihw	(Ignatiadis et al., 2016)	https://bioconductor.org/packages/release/bioc/html/IHW.html
Apeglm	(Zhu et al., 2019)	https://bioconductor.org/packages/release/bioc/html/apeglm.html
FastQC	Babraham bioinformatics	http://www.bioinformatics.babraham.ac.uk/projects/fastqc/
Trimmomatic	(Bolger et al., 2014)	http://www.usadellab.org/cms/?page=trimmomatic
Tophat2	(Kim et al., 2013)	https://ccb.jhu.edu/software/tophat/index.shtml

RESOURCE AVAILABILITY

Lead contact

Further information and requests for resources and reagents should be directed and will be fulfilled by the lead contact, Anna Elisabetta Salcini (lisa.salcini@bric.ku.dk).

Materials availability

All unique/stable reagents generated in this study are available from the lead contact without restriction.

Data and code availability

- RNA-seq and ChIP-seq data are deposited to NCBI GEO under the GEO number: GSE174652.
- This paper does not report original code
- Any additional information required to reanalyze the data reported in this paper is available from the lead contact upon request

EXPERIMENTAL MODEL AND SUBJECT DETAILS

Animals

C. elegans strains were cultured according to standard procedure (Brenner, 1974). Animals were grown on Nematode Growth Medium (NGM) seeded with *E. coli* OP50. Experiments were performed at 20°C unless otherwise indicated in the legends of the figure and in Methods details. A list of strains used in this study is provided in Key resources table. Note that *jmjd-5(zr1234)* corresponds to *jmjd-5(DD)*, previously described (Amendola et al., 2017). Compound mutants were generated by standard crossing procedures. *jmjd-5(tm3735)* and *jmjd-5(zr1234)* were backcrossed at least 7 times and one time before starting a new experiment. Other strains were backcrossed at least 3 times before use. As *utx-1(tm3118)* animals are sterile, we used a *utx-1* null allele expressing under its own promoter, a catalytic-dead version of *utx-1*, that re-establishes the viability and fertility of *utx-1* mutants, despite an increased methylation level of H3K27 (Vandamme et al., 2012). For *jmjd-5::GFP,* the allele *jmjd-5(syb1946)* was constructed by SunyBiotech (https://www.sunybiotech.com) using CRISPR-Cas9 technology. The *syb1946* allele has an insertion of GFP construct before the first start codon of the *jmjd-5* gene. sgRNA target sites are sg1: AAAATTGACGAGTGTCGCGACGG sg2: TATTCGAAGAGCAAAACTG TCCGG.

The experimental protocols used in this work do not require an ethic statement.

METHOD DETAILS

Brood size

Worms were singled out and the progeny counted. Broodsize of a strain is represented as the average of all individual broodsizes ± SEM. For broodsize experiments at 25°C, 15-20 L4 were moved from 20°C to 25°C and the progeny of these animals was considered F1.

Mortal germline assay

8 lines of each genotype were established by singling out L1 animals from a mixed population plate. Between generations, 6 L4 of each line were transferred in a plate and a line was defined sterile when no progeny was produced. Progeny of the L1 singled out to create the lines are considered F1.

Endomitotic eggs and germline length

Detection of endomitotic eggs was done 24 hours after L4 and measurement of germline length was performed at early L4 stage. Animals were stained with DAPI and analyzed using the microscope ZEISS Axio Imager. Pictures of endomitotic eggs were acquired at 40X. Germline length was measured using ImageJ (ImageJ, National Institute of Health, Bethesda, MD) (Fiji) and images acquired at 10X. Three biological replicas were used for the analyses.

Reversibility assay

L4 animals were moved from 20°C to 25°C and grown until a strong reduction of fertility was observed at 25°C in *jmjd-5* mutants. L1 larvae of *jmjd-5(zr1234)* and *jmjd-5(tm3735)* grown 25°C for five and six generations, were singled out and kept at 25°C or shifted to 20°C. Broodsizes at 25°C and 20°C were calculated at the indicated generations. Two biological replicas were used for the analysis.

Trans-differentiation assay

L4 animals were kept at 15°C or moved from 15°C to 25°C and grown to the indicated generations. Gonads of young adult hermaphrodites were dissected (24h post L4, unless stated otherwise) by placing animals on a poly-lysine slide containing 8 μL sodium azide (1:50) and cut underneath the pharynx. A cover slide was placed on top and slides brought to the fluorescence microscope (ZEISS Axio Imager) for analysis. Dissected gonads were scored positive if a clear GFP-signal was detected in the extracted germline. Images of entire gonads were acquired at 20X and of single cells at 100X. Investigators were not blinded during the experiments.

Germ cell apoptosis assay

Adults (24 hours post L4) were irradiated and, after 24 hours incubated in for 4 hours at room temperature in 33 mM aqueous solution of SYTO12. Worms were left for 45 min. on OP50 plates to recover and germlines were scored for the presence of fluorescent apoptotic cells.

Immunofluorescence

Embryos were extracted by cutting adult worms (24h post L4) on poly-lysine slides (Thermo Scientific) using a needle. Embryos were then freeze-cracked in liquid nitrogen and incubated with cold methanol for 2 minutes and acetone for 10 minutes. After 5 minutes of PBST wash, blocking was performed for 1 hour in 1% BSA in PBST. Slides were incubated with primary antibodies overnight at 4°C in a humid chamber, washed 4 times for 15 minutes in PBST and incubated for 2 hours at room temperature with secondary antibodies. Gonad of adult hermaphrodites (24h after L4) were dissected on polysine slides (Thermo Scientific) and stained as previously described (Amendola et al., 2017). Briefly, germlines were fixed for 10 minutes with 4% paraformaldehyde and instantly freeze-cracked on dry ice. Gonads were incubated in cold methanol (Merck) for 2 minutes. After 10 minutes of PBST wash, blocking was performed for 1 hour in 1% BSA in PBST. Slides were incubated with primary antibodies overnight at 4°C in a humid chamber. After 3 washes in PBST for 5 minutes, slides were incubated 2 hours with secondary antibodies at room temperature. Slides of embryos and germlines were washed 3 times in PBST before being mounted on coverslips with (Calbiochem 475904). In the second wash, DAPI (Sigma Aldrich D9542) was added at a final concentration of 0.1 ng/ml. Primary and secondary antibodies were diluted in blocking solution as follows: PGL-1 antibody (DSHB K76) 1:10 (Strome and Wood, 1983), H3K27me3 antibody (Millipore 07-449) 1:200, Alexa fluor 568 anti-rabbit (Life technologies A11036) 1:200.

IF Imaging and quantification

Immunofluorescence images were collected at 0.2 μm intervals using a Delta vision platform (GE Healthcare) with IX-71 Olympus microscope Plan Apochromat, LED based 7 color fluorescence illumination module (Lumencor) as light source, CoolSnap HQ2 camera (Photometrics), quadruple filter sets for DAPI, FITC, TRITC, and Cy5. Images were collected using a 100X oil objective (Olympus, 1.4NA) and three-dimensional datasets were subjected to deconvolution using softWoRx 6.5.2.Software Suite (Applied Precision) and then projected onto one dimension using ImageJ (ImageJ, National Institute of Health, Bethesda, MD) (Fiji). Exposure conditions were kept constant for each experiment. Quantification of PGL-1 volumes and intensity in N2 and *jmjd-5(zr1234)* embryos was performed with the Volocity software. Quantification of the H3K27me3/nucleus in N2 and *jmjd-5(zr1234)* germlines was performed using ImageJ (Fiji), by measuring the intensity of nuclei after having subtracted the background. For *jmjd-5::GFP* images, germlines were extracted in M9 on poly-lysine slides and images acquired using Zeiss Axio Imager and 60X oil objective. Exposure conditions were kept constant throughout the acquisition of images of the entire germline. Quantifications were performed using slides produced in at least two independent biological replica.

Germline RNA sequencing

Single germlines from N2 and *jmjd-5(zr1234)* were extracted from adult hermaphrodites (24 hours post L4) grown at the indicated temperature and generations and collected using an aspirator tube (Sigma-Aldrich A5177-5EA). RNA was isolated from single germlines (5 germlines each strain) using Arcturus PicoPure RNA isolation kit (Appliedbiosystem 12204-01) and RNase-Free DNase Set (QIAGEN 79254). Sequencing libraries were constructed using TruSeq RNA Library Prep Kit v2 (Illumina, RS-122-2001) and their quality and size checked with Bioanalyzer (Agilent 2100 Bioanalyzer). Libraries were sequenced using a NextSeq 500 system and a NextSeq 500/550 High Output Kit v2 (Illumina, FC-404-2005). Gene Ontology (GO) analysis was carried out using DAVID (Database for Annotation, Visualization and Integrated Discovery). p value of each enriched GO-category was adjusted using Bonferroni adjustment and presented as $-\log_{10}$(p value). DE genes used for GO-analysis had p value < 0.01.

Classification of DE genes in categories

The expected number of genes in each category was calculated as followed:

$$\text{Expected number of genes} = (\text{Genes in a category} / \text{total number of genes}) \times \text{up/down regulated DEgenes}$$

The expected number of genes in each category was compared to the observed number of genes using Fisher Exact test to test for statistically significant differences. Total number of germline-expressed genes (average of at least 2 reads) is 10,561 at 25°C. *Germline Expressed* category consist of 4699 genes and *Germline Enriched* category of 1063 genes (Wang et al., 2009). Genes belonging to the soma-specific category were derived from (Knutson et al., 2017).

X chromosome to Autosome ratio

Chromosomal location of DE gene was found using the biomart tool from ensembl.org and the number of X located genes divided by the total number of DE genes found on autosomes.

DE genes comparison to other studies

DE genes were compared with several published gene lists (see below). Expected number of overlapping DE genes was calculated as described earlier, and Fisher Exact Test was carried out to test for significant differences between expected and observed overlap. P value < 0.05 was used as significance overlap. DE genes in PGL-1 mutants are from (Knutson et al., 2017), DE genes in MES-2 mutants are from (Seelk et al., 2016) (using FC < −0.4 for downregulated genes and FC > 0.5 for upregulated genes), DE genes in MES-4 mutants are from (Tabuchi et al., 2014), Germline Expressed and Enriched genes were from (Wang et al., 2009), Soma specific genes were from (Knutson et al., 2017) and Oogenic genes were from (Ortiz et al., 2014).

Whole worm RNA sequencing

Young adults (45-48 hours after embryos hatching) grown at 25°C for three generations (F3) were collected and washed in M9 to remove residual bacteria, flash-frozen in liquid nitrogen and stored at −80°C before RNA extraction. RNA was isolated from 4 independent cultures for each strain using TRIzol reagent (Life Technologies) and RNase-Free DNase Set (QIAGEN 79254). Sequencing libraries was constructed using TruSeq RNA Library Prep Kit v2 (Illumina, RS-122-2001) and their quality and size checked with Nano assay at Bioanalyzer (Agilent 2100 Bioanalyzer). Libraries were sequenced using a NextSeq 500 system and a NextSeq 500/550 High Output Kit v2 (Illumina, FC-404-2005). Gene Ontology (GO) analysis was carried out on differentially expressed genes using DAVID (Database for Annotation, Visualization and Integrated Discovery. The p value of each enriched GO-category was adjusted using Bonferroni and presented as $-\log_{10}$(p value). DE genes used for GO-analysis had p value < 0.01 and Log2fC ± 1.

Chromatin immunoprecipitation (ChIP)

N2 and *jmjd-5(zr1234)* young adult worms were collected at the indicated temperature and generations, washed 3 times in M9 and flash-frozen in liquid nitrogen. Frozen drops were grinded to powder using cold mortar and pestle, and the powder is then incubated with 1% formaldehyde (diluted in M9) for a final volume of 15 mL each tube and gently rocked for 10 minutes at room temperature. Crosslinking is stopped adding 0.125 mM Glycine. Crosslinked lysate was centrifuged 3 times (4000 G, 3′) and washed in PBS containing inhibitors of proteases. The lysate was resuspended with 10ml of ice cold SDS-buffer (100mM NaCl, 1M Tris-HCl pH 8.1, 0.5M pH 8, 0.02% NaN3, 10% SDS). After centrifugation (2000 G, 10 minutes) supernatant was removed and the pellet resuspended with IP buffer (2:1 ratio of SDS-buffer:Triton dilution buffer, 1M Tris-HCl pH 8.6, NaCl 5M, 0.5M EDTA pH 8, 0.02% NaN3, 5% Triton X-100). Chromatin was sonicated (10-15 cycles, 30″on 30″off, high sonication) using Diagenode bioruptor sonicator. Centrifugation (20000 G 30 minutes) was performed and supernatant collected. Chromatin was de-crosslinked for 3h at 65°C (1% SDS, 0.1M NaHCO3) and chromatin fragments size estimated in 1.5% agarose gel. Protein concentration was calculated using Bradford assay. IP was performed using 250-300ug of protein each sample and *Drosophila* spike-in was included in the IP with 1:10 ratio. Pre-cleared chromatin was incubated ON at 4°C with 3ug of antibody. Antibodies used for ChIP are the following: H3K27me3 antibody (MABI0323), H3K36me2 antibody (abcam 9049), H3K36me3 antibody (Active motif 61902). Immunoprecipitated complexes were recovered on magnetic protein G Dynabeads (Invitrogen) for 2-3h. Dynabeads were washed using low salt (1% Triton X-100, 0.1% SDS, 150mM NaCl, 2mM EDTA pH 8, 20mM Tris-HCl pH 8) and high salt (1% Triton X-100, 0.1% SDS, 500mM NaCl, 2mM EDTA pH 8, 20mM Tris-HCl pH 8) buffers and the de-crosslinking performed ON at 65°C (1% SDS, 0.1M NaHCO3). DNA was purified with PCR MinElute PCR purification kit (QIAGEN 28006) and DNA concentration measured using qubit assay. Libraries for sequencing were prepared using NEBNext Ultra II DNA Library Prep Kit for Illumina (NEB E7645S) and Multiplex Oligos for Illumina (NEB E7335S, E7500S). Quality and size of the libraries were checked by Bioanalyzer (Agilent 2100 Bioanalyzer). Libraries were sequenced using a NextSeq 500 system and a NextSeq 500/550 High Output Kit v2 (Illumina, FC-404-2005).

Bioinformatic analyses

For ChIP-seq, fastq reads were aligned to *C. elegans* and *Drosophila* reference genomes (downloaded from wormbase, PRJNA13758, and UCSC, dm6, respectively) using bowtie1 (Langmead, 2010) with default parameters, discarding multimapping reads. PCR duplicates were removed using samblaster (Faust and Hall, 2014). Reads aligning to both PRJNA13758 and dm6 genomes were removed. Peaks were called with MACS2 (Zhang et al., 2008) with a p value threshold of 1e-5 and were further annotated with ChIPseeker (Yu et al., 2015). To eliminate differences in library size and to perform a calibrated quantification of ChIP-seq signal, we calculated a normalization factor based on the reads aligning to the spike-in genome as described in (Orlando et al., 2014). To correct any potential variability caused by the spike-in mixing, these normalization factors were further corrected based on the ratios of spike-in reads and *C.elegans* reads from the input samples. Bam files were transformed to bigwig files using the function bamCoverage from deepTools (Ramírez et al., 2016) by applying the normalization factors obtained from the previous step in the –scaleFactor parameter. The formula used to calculate the normalization factors is:

$$Normalization\ factor = \frac{1}{N\ spike\ reads\ in\ ChIP} \times \frac{N\ spike\ reads\ in\ Input}{N\ c.\ elegans\ reads\ in\ Input}$$

Heatmaps and intensity plots of ChIP-seq signal across peaks were performed with computeMatrix and plotHeatmap from deepTools (Ramírez et al., 2016). Genome-wide and on gene body quantification of ChIP-seq signal were performed with deeptools (Ramírez et al., 2016) with the function multiBigWigSummary bins (5kb), for genome-wide quantification, and multiBigWigSummary bed-file (gene bodies bed as input, downloaded from UCSC, ce10 assembly), for on gene body quantification. To define the

consensus set of H3K36me2 and H3K27me3 peaks, we merged all the peaks from N2 and jmjd-5 samples at 20°C that were called by MACS2. From that list of peaks, we obtained a list of genes with at least 1 peak falling between their TSS and TES. We then quantified H3K36me2 and H3K27me3 signal across those gene bodies, and used this data to cluster these targets in 5 different groups by k-means clustering. This allowed us to distinguish regions highly enriched in H3K36me2 or H3K27me3, and regions enriched in both histone marks.

For the RNA-seq of whole worm analyses, reads were aligned to *C.elegans* reference genome, ce10, using STAR (Dobin and Gingeras, 2015) with default parameters and removing multimapping reads. PCR duplicates were removed with samblaster (Faust and Hall, 2014) and gene quantification was performed using featureCounts (Liao et al., 2014) with default parameters. Differential expression analyses were performed with the R package DESeq2 (Love et al., 2014). P values and log2 fold changes were further corrected with ihw (Ignatiadis et al., 2016) and apeglm (Zhu et al., 2019) R packages, respectively, with default parameters. Finally, genes with an adjusted p value < 0.01 and absolute log2FC $> = 1$ were considered differentially expressed.

For the germline RNA-seq, the quality of the RNA reads was assessed using

FastQC (https://www.bioinformatics.babraham.ac.uk/projects/fastqc/). The reads were trimmed, filtered with a Phred quality score of at least 25 and all adapters removed with Trimmomatic software (Bolger et al., 2014).

Clean reads were aligned versus the N2 *Caernohabditis elegans* reference genome (release WBcel235.85, http://useast.ensembl.org/Caenorhabditis_elegans/Info/Index) by using Tophat2 (Kim et al., 2013) with default parameters. After counting reads per gene with the FeatureCounts tool (Liao et al., 2014), quantitative differential expression analysis between conditions was performed by DESeq2 (Love et al., 2014). To control the False Discovery Rate (FDR), p values were amended by Benjamini-Hochberg (BH) multiple testing correction (Benjamini, 1995).

QUANTIFICATION AND STATISTICAL ANALYSIS

p values were calculated using GraphPad Prism and values and asterisks are stated in figure legends. Error bars designate standard error of the mean (SEM). For RNaseq the p values of DEgenes were subjected to Benjamini-Hochberg (BH) multiple testing correction. p values of enriched GO-terms were adjusted using Bonferroni correction. One-way ANOVA, Student t test, Fisher Exact test were used to analyze the data and asses statistical significance, the test used to analyze the data in question is stated in figure legends. Significance of overlaps between lists of genes was assessed using Fisher Exact test. Asterisks in the figures indicate significance as following: * $p < 0.05$; ** $p < 0.01$; *** $p < 0.001$; **** $p < 0.0001$.

Cell Reports, Volume *37*

Supplemental information

Coordinated maintenance of H3K36/K27 methylation by histone demethylases preserves germ cell identity and immortality

Nico Zaghet, Katrine Madsen, Federico Rossi, Daniel Fernandez Perez, Pier Giorgio Amendola, Samuel Demharter, Ulrich Pfisterer, Konstantin Khodosevich, Diego Pasini, and Anna Elisabetta Salcini

Figure S1

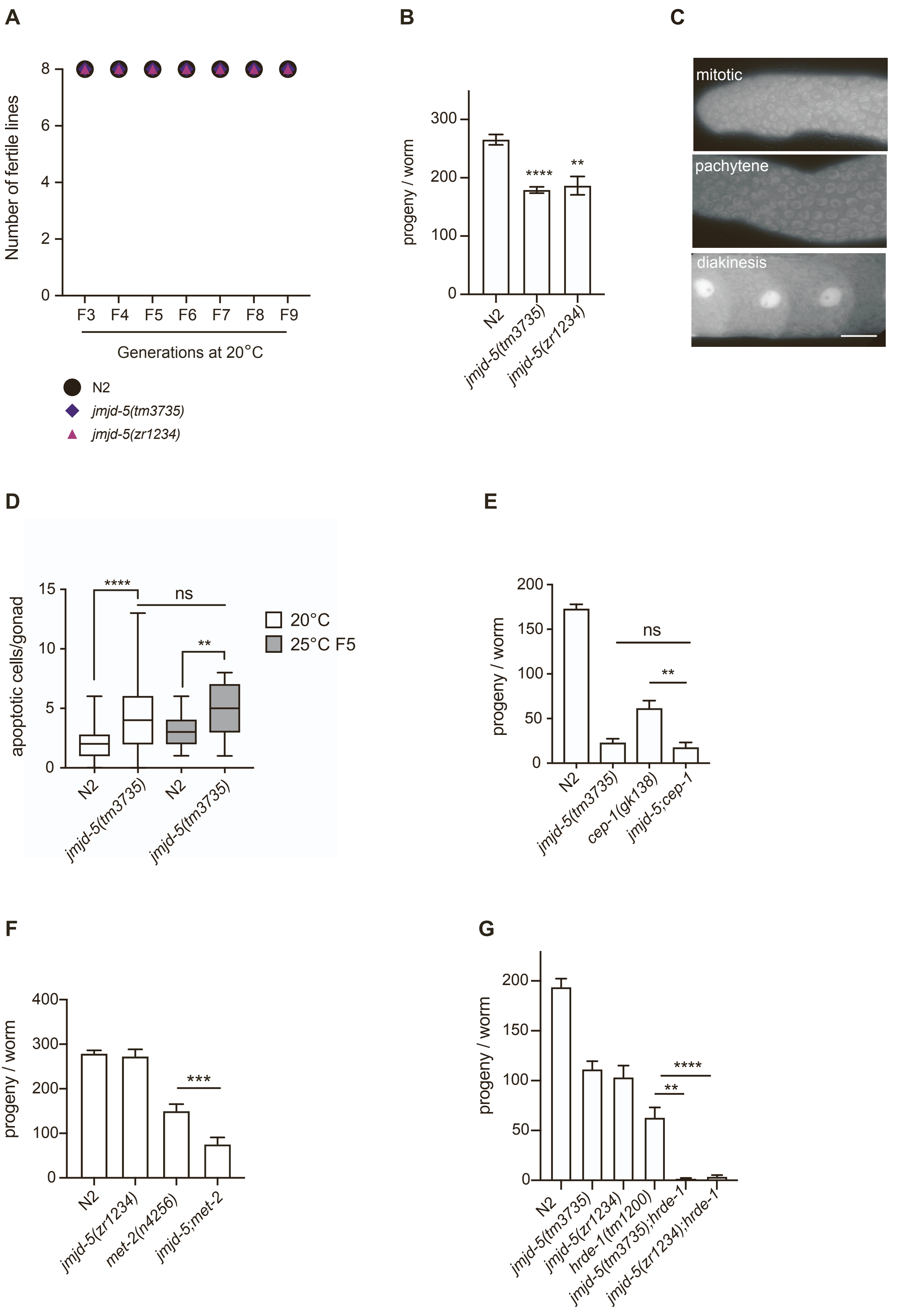
A
B
C
Number of fertile lines
8
6
4
2
0
F3 F4 F5 F6 F7 F8 F9
Generations at 20°C
N2
jmjd-5(tm3735)
jmjd-5(zr1234)
progeny / worm
300
200
100
0
****
**
N2
jmjd-5(tm3735)
jmjd-5(zr1234)
mitotic
pachytene
diakinesis
D
E
apoptotic cells/gonad
15
10
5
0
****
ns
**
20°C
25°C F5
N2
jmjd-5(tm3735)
N2
jmjd-5(tm3735)
progeny / worm
200
150
100
50
0
ns
**
N2
jmjd-5(tm3735)
cep-1(gk138)
jmjd-5;cep-1
F
G
progeny / worm
400
300
200
100
0
***
N2
jmjd-5(zr1234)
met-2(n4256)
jmjd-5;met-2
progeny / worm
200
150
100
50
0
****
**
N2
jmjd-5(tm3735)
jmjd-5(zr1234)
hrde-1(tm1200)
jmjd-5(tm3735);hrde-1
jmjd-5(zr1234);hrde-1

Figure S1. *jmjd-5* preserves the functionality of the germ cells at high temperature, independently of its role in DNA damage response (Related to Fig.1)

(A) Mortal germline assay of *jmjd-5(zr1234)* and *jmjd-5(tm3735)* at 20°C. n=8. (B) Brood size of N2, *jmjd-5(zr1234)* and *jmjd-5(tm3735)* at 15 °C. n>11. (C) Representative image of *jmjd-5::GFP* expression in adult germ cells, detected by direct fluorescence. Bar is 20μm. (D) Boxplot showing the quantification of apoptotic germ cells, using SYTO-12 staining, in N2 and *jmjd-5(tm3735)* grown at 20°C or 25°C for five generations (F5). n>20. (E) Brood size of N2, *jmjd-5(tm3735), cep-1(gk138)* and double mutant grown at 25°C for eight generations (F8). n=20. (F). Brood size of N2, *jmjd-5(zr1234), met-2(n4256)* and double mutants at 20°C. n>10. (G) Brood size of N2, *jmjd-5(zr1234), jmjd-5(tm3735), hrde-1(tm1200)* and double mutants at F1 generation at 25°C. n>10. In B, D-G, bars indicate SEM. ****p<0.0001, ***p<0.001, **p<0.01, ns= not significant, with one-way ANOVA.

Figure S2

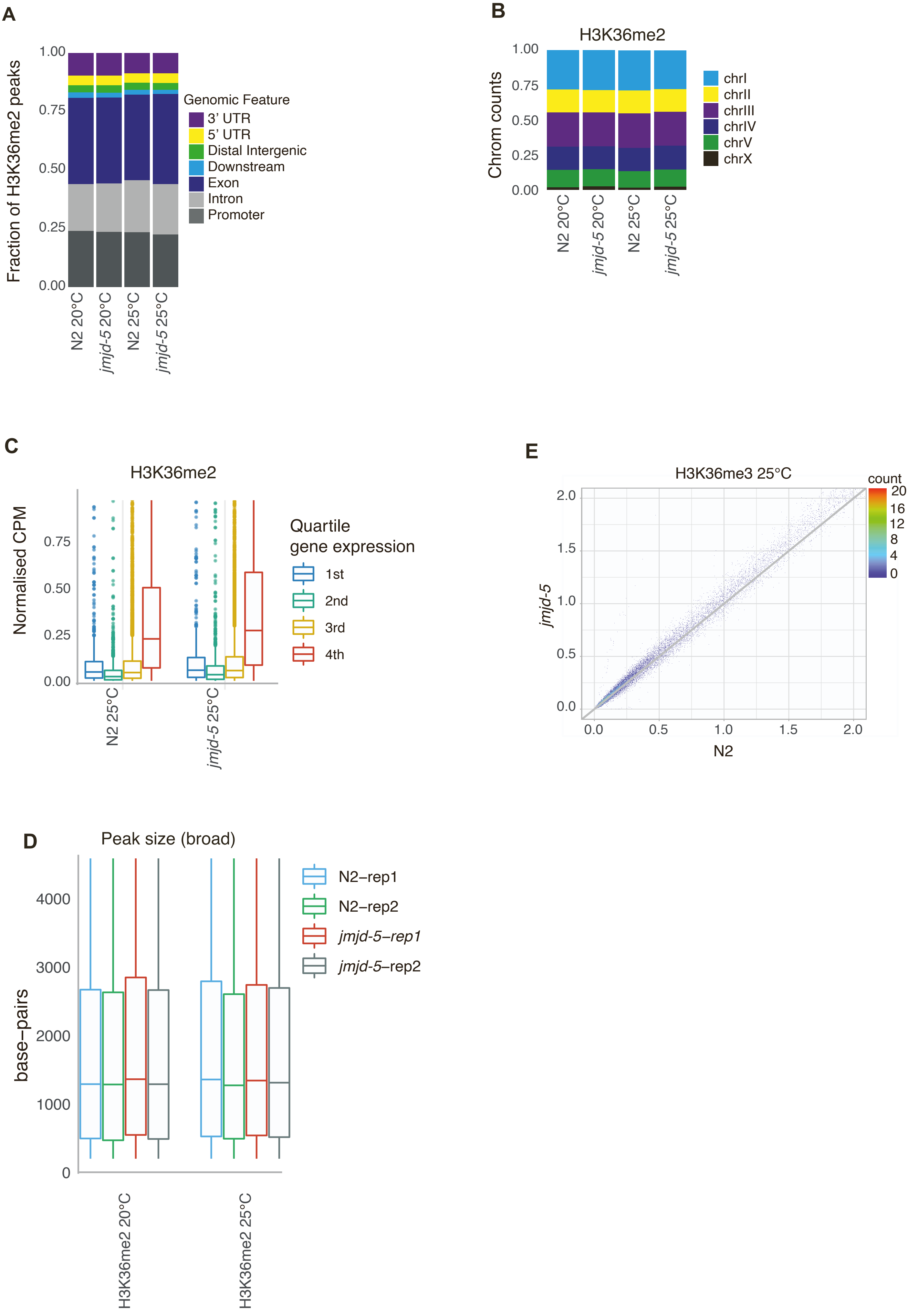

A
Fraction of H3K36me2 peaks
1.00
0.75
0.50
0.25
0.00
N2 20°C
jmjd-5 20°C
N2 25°C
jmjd-5 25°C
Genomic Feature
3' UTR
5' UTR
Distal Intergenic
Downstream
Exon
Intron
Promoter
B
H3K36me2
Chrom counts
chrI
chrII
chrIII
chrIV
chrV
chrX
C
H3K36me2
Normalised CPM
0.75
0.50
0.25
0.00
N2 25°C
jmjd-5 25°C
Quartile
gene expression
1st
2nd
3rd
4th
E
H3K36me3 25°C
jmjd-5
N2
count
20
16
12
8
4
0
2.0
1.5
1.0
0.5
0.0
D
Peak size (broad)
base−pairs
4000
3000
2000
1000
0
H3K36me2 20°C
H3K36me2 25°C
N2−rep1
N2−rep2
jmjd-5−rep1
jmjd-5−rep2

Figure S2. H3K36me2/3 ChIP analyses (Related to Fig.4)

(A) Genomic distribution of H3K36me2 peaks in genomic features. Promoters are considered as +/-100bp around TSS, Downstream is considered as > 0 and <1000bp from TES (Transcription End Site) and Distal intergenic +1000bp from TSS (Transcription Start Site). (B) Proportion of H3K36me2 reads aligned to each chromosome in the represented samples. (C) H3K36me2 ChIP-seq signal across all *C. elegans* protein coding genes, divided in 4 quartile groups based on their mRNA expression levels in N2 25°C samples. (D) Peak-size distribution of H3K36me2 ChIP-seq samples. Data for both biological replicates are displayed. (E) Scatterplot of H3K36me3 5kb genome-wide bins signal. N2 vs *jmjd-5(zr1234)* at 25°C is shown.

Figure S3

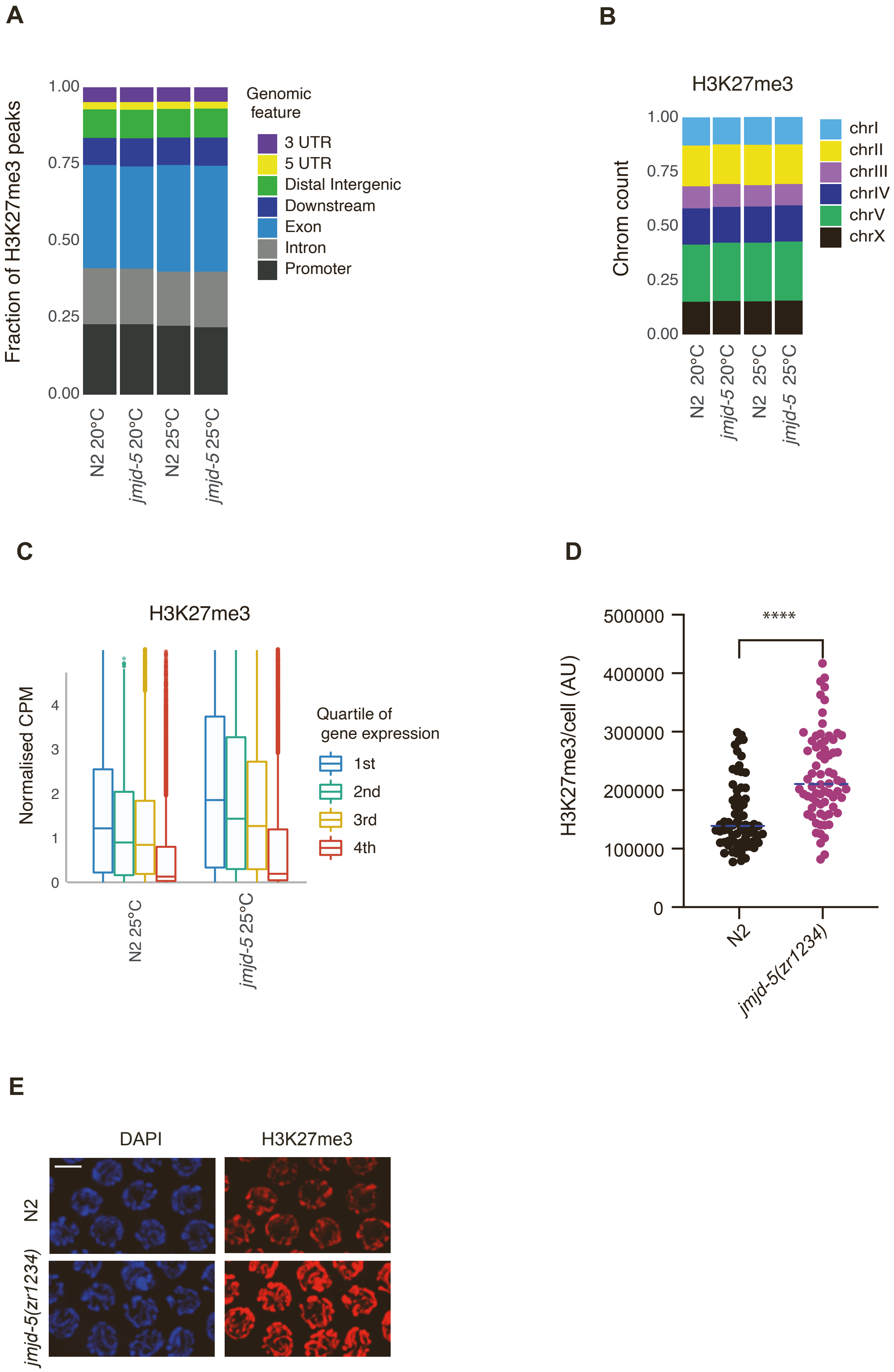
A
Fraction of H3K27me3 peaks
1.00
0.75
0.50
0.25
0.00
N2 20°C
jmjd-5 20°C
N2 25°C
jmjd-5 25°C
Genomic feature
3 UTR
5 UTR
Distal Intergenic
Downstream
Exon
Intron
Promoter
B
H3K27me3
Chrom count
1.00
0.75
0.50
0.25
0.00
N2 20°C
jmjd-5 20°C
N2 25°C
jmjd-5 25°C
chrI
chrII
chrIII
chrIV
chrV
chrX
C
H3K27me3
Normalised CPM
0
1
2
3
4
N2 25°C
jmjd-5 25°C
Quartile of gene expression
1st
2nd
3rd
4th
D
H3K27me3/cell (AU)
500000
400000
300000
200000
100000
0
****
N2
jmjd-5(zr1234)
E
DAPI
H3K27me3
N2
jmjd-5(zr1234)

Figure S3. H3K27me3 ChIP and IF analyses (Related to Fig. 5)

(A) Genomic distribution of H3K27me3 peaks. Promoters are considered as +/-100bp around TSS (Transcription Start Site), Downstream is considered as > 0 and <1000bp from TES (Transcription End Site) and Distal intergenic +1000bp from TSS. (B) Proportion of H3K27me3 reads aligned to each chromosome in the represented samples. (C) H3K27me3 ChIP-seq signal across all *C. elegans* protein coding genes, divided in 4 quartile groups based on their mRNA expression levels in N2 25°C samples. (D) Quantification of the average H3K27me3 intensity (in arbitrary units) per nucleus in pachytene region of N2 and *jmjd-5(zr1234)*. At least 70 cells derived from 7 germlines were quantified for each strain. Lines represent mean. ****$p<0.0001$ with two tailed unpaired t-test. (E) Representative image of H3K27me3 staining in the pachytene region of N2 and *jmjd-5(zr1234)*.

Figure S4

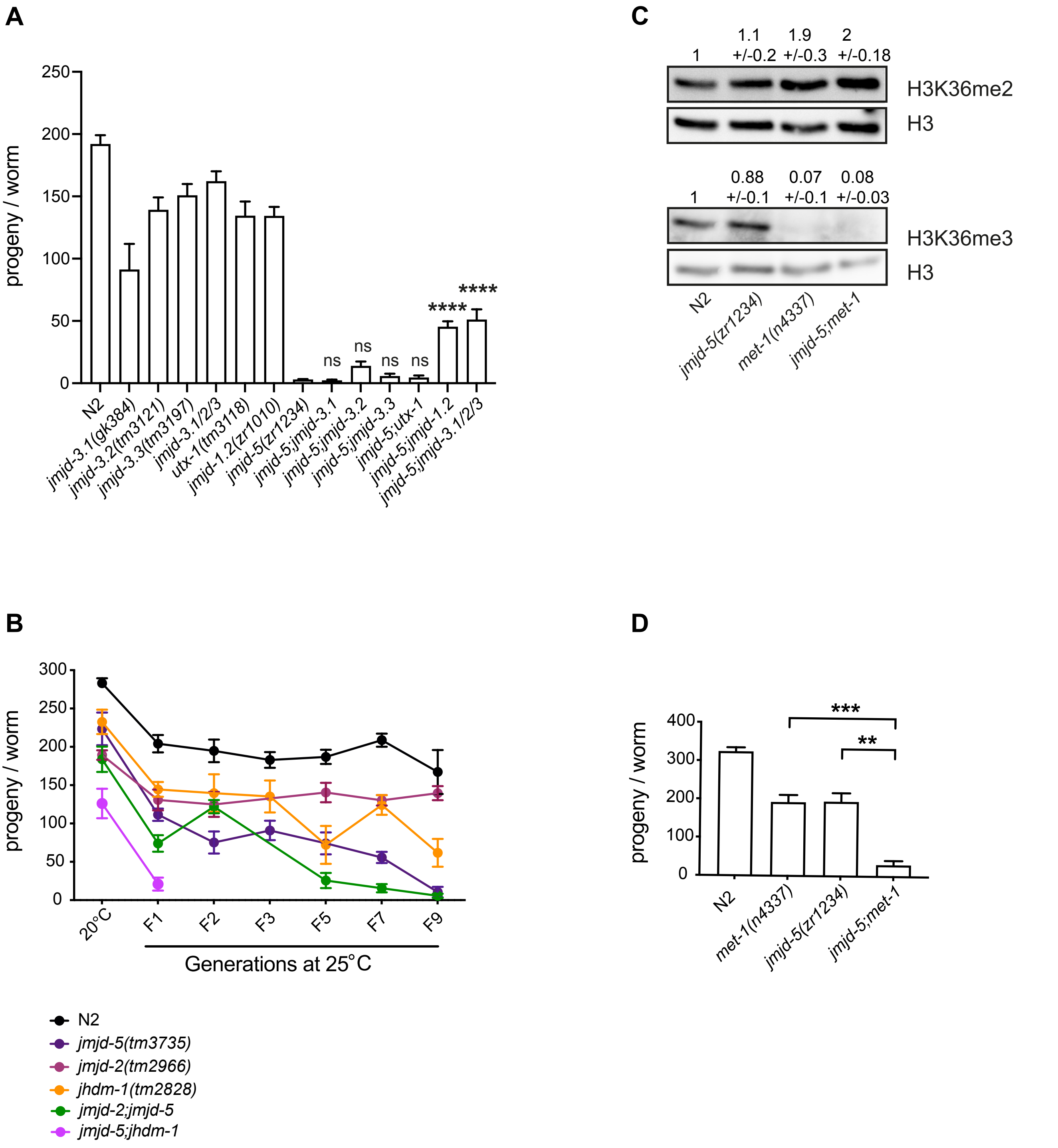
A
progeny / worm
250
200
150
100
50
0
ns
ns
ns
ns
****
****
N2
jmjd-3.1(gk384)
jmjd-3.2(tm3121)
jmjd-3.3(tm3197)
jmjd-3.1/2/3
utx-1(tm3118)
jmjd-1.2(zr1010)
jmjd-5(zr1234)
jmjd-5;jmjd-3.1
jmjd-5;jmjd-3.2
jmjd-5;jmjd-3.3
jmjd-5;utx-1
jmjd-5;jmjd-1.2
jmjd-5;jmjd-3.1/2/3
C
1
1.1 +/-0.2
1.9 +/-0.3
2 +/-0.18
H3K36me2
H3
1
0.88 +/-0.1
0.07 +/-0.1
0.08 +/-0.03
H3K36me3
H3
N2
jmjd-5(zr1234)
met-1(n4337)
jmjd-5;met-1
B
progeny / worm
300
250
200
150
100
50
0
20°C
F1
F2
F3
F5
F7
F9
Generations at 25°C
N2
jmjd-5(tm3735)
jmjd-2(tm2966)
jhdm-1(tm2828)
jmjd-2;jmjd-5
jmjd-5;jhdm-1
D
progeny / worm
400
300
200
100
0
***
**
N2
met-1(n4337)
jmjd-5(zr1234)
jmjd-5;met-1

Figure S4. Genetic interaction of *jmjd-5* with H3K36/K27 methylation regulators (Related to Fig. 7)

(A) Brood size of N2 and indicated mutant strains grown at 25°C for seven generations. Bars represent SEM. n>10. ****p<0.0001, ns, not significative with one-way ANOVA multiple comparison to *jmjd-5(zr1234)*. *utx-1(tm3118)+UTX-1DD::GFP* expresses, under an endogenous promoter, a catalytic-dead version of *utx-1*. (B) Brood size of N2 and indicated mutant strains grown at 20°C and at 25°C, at the indicate generations. (C) Representative western blot and quantified level of H3K36me2/3 (relative to N2), using lysates from synchronized young adult of the indicated strains. H3 is used as loading control. Quantification was performed using two biologically independent replica. (D) Brood size of N2 and indicated mutant strains at 20°C. In A, B and D bars represent SEM. n>15. **** p<0.0001, ***p<0.001, **p<0.01, ns= not significant, with two tailed unpaired t-test.

Figure S5

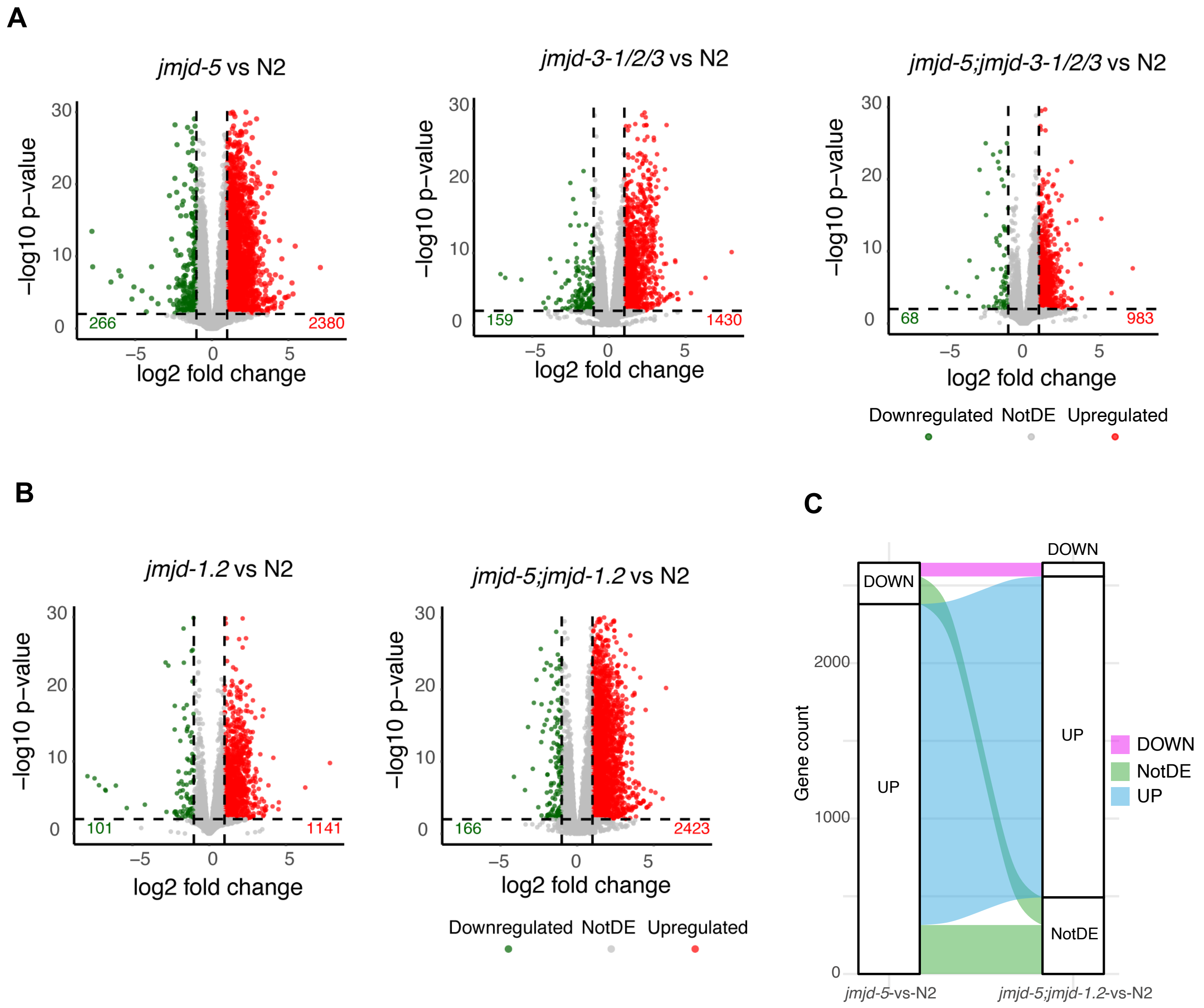
A
jmjd-5 vs N2
jmjd-3-1/2/3 vs N2
jmjd-5;jmjd-3-1/2/3 vs N2
−log10 p−value
log2 fold change
266
2380
159
1430
68
983
Downregulated NotDE Upregulated
B
jmjd-1.2 vs N2
jmjd-5;jmjd-1.2 vs N2
101
1141
166
2423
C
DOWN
UP
NotDE
Gene count
jmjd-5-vs-N2
jmjd-5;jmjd-1.2-vs-N2

Figure S5. Analyses of differentially expressed genes in *jmjd-5(zr1234)*, H3K27 demethylases mutants and compound mutants (Related to Fig. 7).

(A) Volcano plots representing the log2FC and -$\log_{10}$(p-adjusted) of total worm RNA-seq resulting from the following comparisons: *jmjd-5(zr1234)* vs N2 (left), *jmjd-3.1/2/3* vs N2 (center) and *jmjd-5;jmjd-3.1/2/3* vs N2 (right). (B) Volcano plots representing the log2FC and -$\log_{10}$(p-adjusted) of total worm RNA-seq resulting from the following comparisons: *jmjd-1.2* vs N2 (left) and *jmjd-5;jmjd-1.2* vs N2 (right). In A and B, genes with adjusted p-value < 0.01 and log2FC >+/- 1 are considered differentially expressed. (C) Alluvial diagram of differentially expressed genes from *jmjd-5(zr1234)* vs N2 (25°C) and *jmjd-5;jmjd-1.2* vs N2 (25°C).

Table S2. ***jmjd.5*** **prevents germ cells trans-differentiation (Related to Fig. 3)**

Genotype	%P0	%F3	%F4
jmjd-5(zr1234);unc-119::GFP (DP132)	0 (0/64)	18.9 (10/53)	29.4 (15/51)
unc-119::GFP(DP132)	0 (0/30)	0 (0/35)	0 (0/33)
jmjd-5(zr1234);unc-75::GFP(OH443)	0 (0/32)	27.3 (9/33)	35.5 (11/31)
unc-75::GFP(OH443)	0 (0/20)	0 (0/31)	0 (0/30)
jmjd-5(zr1234);lin-17::GFP (KS411)	5.4 (3/56)	33.3 (18/54)	41.9 (14/31)
lin-17::GFP(KS411)	0 (0/31)	0 (0/50)	0 (0/53)

Table S2. Trans-differentiation assay

Extracted germlines of adult animals of the indicated genotypes were analyzed for the presence of GFP fluorescence at the indicated generations at 25°C. n>30
